# Genetic Structure and Linkage Disequilibrium in a Diverse, Representative Collection of the C4 Model Plant, *Sorghum bicolor*

**DOI:** 10.1534/g3.112.004861

**Published:** 2013-05-01

**Authors:** Yi-Hong Wang, Hari D. Upadhyaya, A. Millie Burrell, Sayed Mohammad Ebrahim Sahraeian, Robert R. Klein, Patricia E. Klein

**Affiliations:** * Department of Biology, University of Louisiana at Lafayette, Louisiana 70504; † Gene Bank, International Crops Research Institute for the Semi-Arid Tropics (ICRISAT), Patancheru 502 324, Andhra Pradesh, India; ‡ Department of Horticultural Sciences and Institute for Plant Genomics and Biotechnology, Texas A&M University, College Station, Texas 77843; § Department of Electrical and Computer Engineering, Texas A&M University, College Station, Texas 77843; ** USDA-ARS, Southern Plains Agricultural Research Center, College Station, Texas 77845

**Keywords:** sorghum, races, SNPs, *Fse*I-assisted genotyping, linkage disequilibrium

## Abstract

To facilitate the mapping of genes in sorghum [*Sorghum bicolor* (L.) Moench] underlying economically important traits, we analyzed the genetic structure and linkage disequilibrium in a sorghum mini core collection of 242 landraces with 13,390 single-nucleotide polymorphims. The single-nucleotide polymorphisms were produced using a highly multiplexed genotyping-by-sequencing methodology. Genetic structure was established using principal component, Neighbor-Joining phylogenetic, and Bayesian cluster analyses. These analyses indicated that the mini-core collection was structured along both geographic origin and sorghum race classification. Examples of the former were accessions from Southern Africa, East Asia, and Yemen. Examples of the latter were caudatums with widespread geographical distribution, durras from India, and guineas from West Africa. Race bicolor, the most primitive and the least clearly defined sorghum race, clustered among other races and formed only one clear bicolor-centric cluster. Genome-wide linkage disequilibrium analyses showed linkage disequilibrium decayed, on average, within 10−30 kb, whereas the short arm of SBI-06 contained a linkage disequilibrium block of 20.33 Mb, confirming a previous report of low recombination on this chromosome arm. Four smaller but equally significant linkage disequilibrium blocks of 3.5−35.5 kb were detected on chromosomes 1, 2, 9, and 10. We examined the genes encoded within each block to provide a first look at candidates such as homologs of *GS3* and *FT* that may indicate a selective sweep during sorghum domestication.

Sorghum [*Sorghum bicolor* (L.) Moench] is a grass of the steppes and savannas of Africa that was domesticated 3000−5000 years ago and spread to diverse climates and geographical locations worldwide (Mann *et al.* 1983; Kimber 2000). The species is diverse owing to the climate and geography in which it evolved and the selection pressure by humans and the environment (Dahlberg *et al.* 2002). Cultivated sorghum taxonomy is based on spikelet and panicle morphology (Harlan and de Wet 1972). Under these specifications, sorghum is divided into five primary races: bicolor, caudatum, durra, guinea, and kafir, as well as 10 intermediate races, derived from intermating of the primary races. This classification has been used by sorghum researchers and is largely supported by genetic evidence with the exception of race bicolor (Deu *et al.* 1994; Perumal *et al.* 2007; Brown *et al.* 2011). The bicolor race has an open panicle and is the most primitive and heterogeneous of the sorghum races. Bicolor is distributed wherever sorghum is grown. Race guinea also has an open panicle and is found in West Africa although it also occurs in East Africa and India (de Wet 1978). Guinea sorghums were selected from bicolor and were probably the first evolved “specialized” grain sorghum (Smith 1995). Kafir is a race strictly of southern Africa with a distribution that is closely associated with the migration and movements of Bantu agriculturalists. Kafirs are high-yielding with semicompact panicles. Durra is the most specialized of the races, displaying a compact panicle and medium-to-larger grain (de Wet and Huckabay 1967). The durra race is abundant in Ethiopia and Sudan and is essentially the only race of sorghum in India. Finally, race caudatum is believed to be a relatively recent race due to its limited distribution from sorghum’s region of initial domestication in Africa. Caudatums are important to modern agriculture due to their high yielding medium-to-large panicles, but the seeds often contain tannins that make flour bitter and dark which may explain their relatively limited distribution (Stemler *et al.* 1975).

Genetic structure for populations composed of different races affects the efficiency of association (or linkage disequilibrium—LD) mapping. Early studies of population structure in sorghum were focused on differentiating the races based on a limited number of genetic markers. Using restriction fragment length polymorphism markers, Deu *et al.* (1994) were able to differentiate the races caudatum, durra, guinea, and kafir but not race bicolor as expected. With the advent of polymerase chain reaction (PCR)-based markers, numerous researchers have examined the classification of sorghum races based on genetic markers (Cui *et al.* 1995; de Oliveira *et al.* 1996; Menkir *et al.* 1997; Djè *et al.* 2000; Dahlberg *et al.* 2002; Ghebru *et al.* 2002; Menz *et al.* 2004; Casa *et al.* 2005; Folkertsma *et al.* 2005; Perumal *et al.* 2007; Ritter *et al.* 2007; Casa *et al.* 2008; Deu *et al.* 2008; Mace *et al.* 2008; Bouchet *et al.* 2012). The use of larger sets of highly informative markers allows the study of genetic structure through model-free (principal component analysis, or PCA) and Bayesian model-based (STRUCTURE) approaches (Pritchard *et al.* 2000). For example, Brown *et al.* (2011) applied 434 markers to an association panel of 216 converted sorghum lines and found that those lines classified as primary races could be separated by race based on PCA or a Bayesian model. Principal components (PCs) 1, 2, and 3 successfully separated the durra, caudatum, kafir, and guinea races but not the bicolor race. Similar results were obtained with STRUCTURE (Brown *et al.* 2011). More recently, Bouchet *et al.* (2012) reported that durra and bicolor types from India and eastern Africa, and caudatum and caudatum-bicolor types from China belong to the same group after genotyping 177 sorghum lines with 713 selected markers based on Bayesian population structure analysis.

Population genetic structure is a critical factor influencing the outcome of association or LD mapping (Gupta *et al.* 2005). In Arabidopsis, LD increased when accessions sampled regionally were used as the mapping population, whereas globally collected samples as the mapping population demonstrated a decrease in LD, suggesting that globally collected samples are more suitable for fine-mapping of phenotypic traits (Bergelson and Roux 2010). In sorghum, Hamblin *et al.* (2005) sequenced six unlinked regions of 40−100 kb in 24 landraces and nine wild sorghums and found that LD largely decays by 10–15 kb. Based on 177 sorghum lines genotyped with 1122 DArT markers, Bouchet *et al.* (2012) indicated that significant LD could be found for 20% of the pairs of markers in the 50- to 100-kb range and for regions spanning 5 Mbp on chromosome 7 and more than 12 Mbp on chromosome 10.

With the advent of genome-wide, sequenced-based genotyping in sorghum, precise and accurate estimations of population structure and LD across the genome are now attainable. Our goal in this research was to use genotyping-by-sequencing technology to reexamine population structure and LD of a mini-core collection developed from the International Crops Research Institute for the Semi-Arid Tropics (ICRISAT) sorghum core collection. This mini-core collection of 242 accessions is composed entirely of landraces from 57 countries, which were selected based on 11 qualitative and 10 quantitative traits (Upadhyaya *et al.* 2009). This mini-core collection is a representative sampling of the ICRISAT core collection of 22,473 landraces, with accessions originating from 76 countries and displaying a full range of phenotypic traits displayed in the core collection (Grenier *et al.* 2001; Upadhyaya *et al.* 2009). The mini-core collection has been used for association mapping in sorghum using a limited number of simple sequence repeat markers (Wang *et al.* 2011; Upadhyaya *et al.* 2012; Wang *et al.* 2012). Here, we re-examine the genetic structure of the ICRISAT mini core collection by using PCA, Bayesian population structure analysis and Neighbor-Joining phylogenetic analysis and subsequently analyze genome-wide LD to gain a greater understanding of the sorghum genome for the benefit of association mapping with 13,390 SNP markers.

## Materials and Methods

### Plant materials

Two hundred forty-two landraces of the sorghum mini-core collection were used for this study. Race and country of origin information were reported previously (Upadhyaya *et al.* 2009). The collection contains accessions of the five primary races (20 bicolors, 39 caudatums, 30 durras, 29 guineas, 21 kafirs) and 10 intermediate races (30 caudatum-bicolors, 19 durra-caudatums, 7 durra-bicolors, 2 guinea-bicolors, 2 guinea-durras, 3 guinea-kafirs, 2 kafir-bicolors, 7 kafir-caudatums, 27 guinea-caudatums, and 4 kafir-durras).

### SNP genotyping

DNA was isolated from either germinating seedlings or frozen leaf tissues using the FastDNA Spin Kit (MP Biomedicals, Solon, OH) and quantified using a fluorometric assay (Qubit; Life Technologies). DNA purity was assessed by obtaining 260/280 ratios and confirmation of an absorbance maximum at 260 nm using the NanoDrop1000 instrument (Thermo Fisher Scientific, Waltham, MA). SNPs were generated using high-throughput sequence analysis of DNA templates from specific sites targeted by restriction endonucleases (Baird *et al.* 2008; Gore *et al.* 2009; Davey and Blaxter 2010; Elshire *et al.* 2011; Nelson *et al.* 2011). Illumina template was prepared using a method developed by Morishige and Mullet (D. T. Morishige and J. E. Mullet, personal communication). Briefly, 500 ng of genomic DNA was digested with *Fse*I (New England BioLabs, Ipswich, MA). Adapters containing 4-bp identifier tags were ligated onto the *Fse*I-cut end of the resulting fragments, and following adapter ligation, the samples were pooled in groups of 48. Pools were precipitated, sheared to 200−600 bp using a Biorupter sonicator (Diagenode, Denville, NJ), size selected (150−250 bp) by agarose gel electrophoresis and subsequently purified by gel extraction (QIAGEN, Valencia, CA). Samples were then treated with *Bst* DNA Polymerase (New England BioLabs) to repair the ends, A-tails were added to the sheared ends using Klenow 3′-5′ exonuclease (New England BioLabs), and the samples blunt-ended using a Quick Blunting Kit (New England BioLabs). After each of the aforementioned steps, a purification step (QIAGEN) was performed. Following end-repair and A-tailing, a second set of adapters was ligated onto the 3′ end of the product with a 5′ identification adapter and a 3′ general adapter. Ligation reactions were cleaned using AMPure XP Reagent (Beckman Coulter, Indianapolis, IN) to remove excess adapter. Subsequently, products were enriched by PCR amplification using Phusion High-Fidelity DNA Polymerase (New England BioLabs) from the adapters using one standard and one biotinylated primer. The desired products were isolated using magnetic Dynabeads M-280 Streptavidin beads (Invitrogen, Grand Island, NY), and the captured double-stranded DNA was denatured at 98° to form single stranded products. The nonbiotinylated product was carried forward through a second round of PCR. The final PCR resulted in double-stranded products that contained the end sequences necessary for bridge-amplification, and samples at 10 nM concentration were submitted for 78-bp single-end sequencing on an Illumina GAIIx (Illumina, San Diego, CA). For each PCR step, a high-fidelity DNA polymerase with 3ʹ−5ʹ proofreading ability and high processivity was used to reduce the introduction of sequencing errors. In addition, the number of PCR cycles was kept as low as possible to obtain the desired quantity of PCR product while limiting the introduction of PCR errors.

Base call conversion and assignment of quality scores was performed using Illumina’s CASAVA_v1.7 software. Sequences were trimmed, sorted by individual 4-bp multiplex identifier (MID) tags, and filtered for 100% identity of the individual MID tag and partial restriction site sequence. The processed reads for each individual accession were aligned to the sorghum genome sequence (Paterson *et al.* 2009) and analyzed for SNPs using the CLC Bio Genomics Workbench software (Version 5.5.1, CLC Bio, Cambridge, MA). Read mapping parameters were set to insertion, deletion and mismatch cost = 3, 50% minimum read length required to match the reference, and a minimum of 90% similarity between the read and the reference sequence. In addition, reads that aligned to more than one position equally were omitted and thus not included in the final mapping. For SNP Detection in the CLC Bio Genomics Workbench, the parameters included a window length of 9, a maximum gap and mismatch count of 4, a minimum quality of the SNP base of 20, a minimum average quality of the nucleotides surrounding the SNP of 15, and a minimum read coverage for a SNP of 6. The stringent minimum read coverage for each SNP was applied as a means of differentiating a sequencing error from a legitimate SNP. Custom perl scripts were used to process the output from the CLC Bio Genomics Workbench for import into STRUCTURE 2.3 (Pritchard *et al.* 2000) and TASSEL 3.0 (Bradbury *et al.* 2007) for downstream analysis. Genomic positions where base calls were scored in at least 25% of the 242 accessions were retained and the missing data were imputed using fastPHASE (Scheet and Stephens 2006). Following imputation, only SNPs with a minor allele frequency greater than 5% were retained for further use. SNPs were named based on the chromosome on which they mapped followed by the physical location in bp (*e.g.*, SNP chr1_46978664, resides on SBI-01 at position 46,978,664 bp).

### Data analysis

PCA was performed using the package prcomp (Becker *et al.* 1988) in R (version 2.15.1, 64 bit; Ripley 2001). SNP data were transformed manually, and no alleles were collapsed together. The 3D scatter plot was produced with an Excel macro Add-In (http://www.doka.ch/Excel3Dscatterplot.htm) using the PC data output. STRUCTURE 2.3 (Pritchard *et al.* 2000) was run with the admixture model, a burn-in period of 50,000 and 5000 Markov Chain Monte Carlo repetitions. The Neighbor-Joining tree was created using 1000 bootstraps in MEGA5 (Tamura and Nei 1993; Tamura *et al.* 2011). LD as measured by *r*^2^ was calculated in TASSEL 3.0 using a sliding window (Bradbury *et al.* 2007; available from http://www.maizegenetics.net/). Local LD analysis on SBI-06 used the full matrix option in TASSEL. The critical value of *r*^2^ was the conventional 0.1 (Remington *et al.* 2001; Nordborg *et al.* 2002; Palaisa *et al.* 2003). Statistical tests for each *r*^2^ were provided by the *p* value calculated in TASSEL. The sorghum genome (MIPS/JGI Sbi1.4) was searched for annotated genes at www.phytozome.net.

## Results and Discussion

### Genotyping the ICRISAT mini-core collection

The 242 accessions from the ICRISAT mini-core collection were genotyped using a genotyping-by-sequencing approach. Sequencing templates were generated using the methylation-sensitive enzyme *Fse*I. This enzyme recognizes the GC-rich sequence GGCCGGCC that is found in or near many nuclear genes but is not found in the sorghum chloroplast genome. Following template library preparation, 48 accessions, each containing a unique 4-bp MID tag, were pooled/lane on the Illumina GAIIx. An average of 504,000 78-bp sequences was obtained from each accession that contained the expected *Fse*I partial restriction site and 4-bp MID tag. Approximately 91% of the reads from each accession mapped uniquely to the sorghum genome reference sequence. The sorghum genome contains ∼23,000 *Fse*I sites based on *in silico* digestion of the reference BTx623 sequence. Therefore, if every recognition site were restricted with *Fse*I, ∼46,000 reads (*i.e.*, a forward and reverse read from each *Fse*I site) would be expected. However, because *Fse*I is sensitive to methylation, it will only restrict nonmethylated recognition sequences. On average, 22,000 unique reads/genotype were obtained representing ∼1.58Mbp of the sorghum genome. This result was not surprising given the fact that a large percentage (∼50%) of sequences that flank *Fse*I sites across the sorghum genome are repetitive and a subset of these will be methylated.

The reads that uniquely mapped to the reference genome were used to identify SNPs using the SNP Detection Tool in the CLC Bio Workbench. The number of SNPs obtained per accession following mapping to the BTx623 sorghum reference sequence ranged from a high of 13,099 to a low of 2093 (avg. number SNPs per accession = 5507; data not shown). Comparison of SNPs among the 242 accessions identified 76,138 SNPs in at least 2 of the 242 accessions examined. Several steps were taken to ensure that a high-quality set of SNPs was obtained. At each PCR step during template preparation, a high-fidelity, proof-reading polymerase with high processivity was used, and the number of cycles was limited to reduce incorporation of sequencing errors during PCR amplification. During SNP calling in the CLC Bio Workbench, a minimum of six reads was required to cover the position of the SNP to be called. We required that base calls (*i.e.*, reference allele, alternative allele(s) or heterozygous alleles) be obtained from at least 25% of the 242 genotypes analyzed before imputation of missing data. To serve as an internal standard for estimating the extent to which sequencing errors would falsely predict a SNP, template was prepared from the reference genotype, BTx623, and included in multiple lanes on the Illumina flow cell. Ninety-five percent of the BTx623 reads mapped uniquely to the BTx623 reference genome and an average of 115 SNPs were detected across the 8 BTx623 samples (range of 76 to 173) analyzed compared with an average of 5507 SNPs identified in the genotypes representing the mini core collection (data not shown). Only 102 SNPs identified in the mini core collection were present in the BTx623 samples indicating that the SNP calling parameters utilized resulted in a false-positive SNP call less than 1% of the time (*i.e.*, 102 of 13,492 SNPs called). Taken together, these results indicate that the library preparation, mapping and SNP detection parameters utilized in the present study identified a high quality set of SNPs for downstream use.

After removal of SNPs that mapped to super_contigs, those that did not map near an *Fse*I site within the BTx623 genome, those with 75% or more missing data across the 242 accessions, those with a minor allele frequency less than 5%, and those that were also called in BTx623, a total of 13,390 SNPs remained with an average of 1339 SNPs per chromosome (from 810 on chromosome 8 to 2168 on chromosome 1; Supporting Information, Table S1). As expected a greater density of SNPs/Mbp were found in the euchromatic arms of each chromosome where gene density is greatest, and fewer SNPs/Mbp were found in the repeat-rich pericentromeric heterochromatic region of each chromosome consistent with these regions having greater levels of methylation and reduced rates of recombination (Kim *et al.* 2005) (Figure S1A). Analysis of the position of each SNP shows the majority (∼72%) are within 1 kbp of an adjacent SNP (Figure S1B).

### Principal component analysis (PCA)

**Figure 1:**
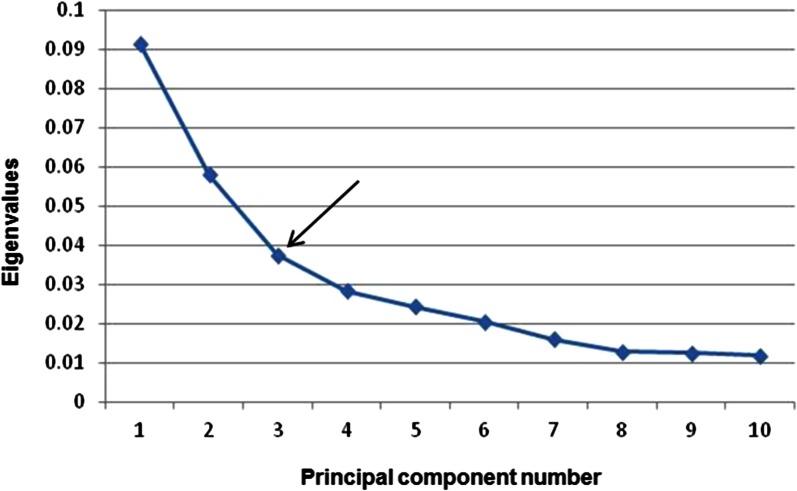
Scree plot of the PCs (X-axis) and their contribution to variance (Y-axis). Arrow indicates the “elbow” point.

The 13,390 SNPs were coded in numbers and used to run prcomp() in R. To determine the number of PCs to use in clustering the mini-core accessions, a scree plot was generated in which the proportion (eigenvalues) of an individual PC’s contribution to total variation was plotted against the number of PCs (Figure 1). The characteristic “elbow” point occurred at 3, indicating that the remaining eigenvalues are relatively small and all about the same size (Figure 1). Therefore, the first three PCs were used in the clustering analysis. These three PCs explained 9.15%, 5.81%, and 3.75% of the total variation identified by the SNP markers. The first 10 PCs together explained 31.32% and the first 24 PCs explained 43.34% of the total variation. The numbers were similar to those reported by Brown *et al.* (2011), who found the first three PCs explained 8.3%, 7.9%, and 4.7% of the total variation using 216 sorghum accessions, respectively.

**Figure 2:**
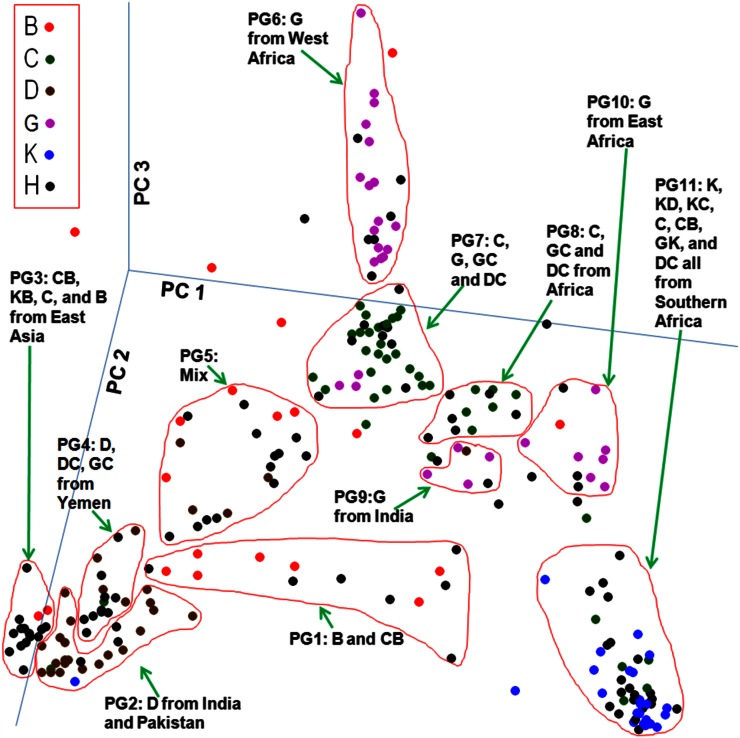
Distribution of sorghum mini-core accessions based on three PCs. Eleven groups were identified based on PC and are labeled PG1 through PG11. B, bicolor; C, caudatum; D, durra; G, guinea, K, kafir, H, hybrid races such as KD, KC, CB, etc. See Table S2 for a list of accessions in each PG.

The first three PCs were plotted in 3D to best capture the distribution of all accessions (Figure 2). The accessions clustered based on race and by geographical origin into 11 PCA groups (PGs). The strongest clustering by geography was with accessions from the southern African countries of Lesotho, Botswana, Zimbabwe, Swaziland, and South Africa. Almost all kafirs (19 of 21) were in this group (PG11), which also included kafir-durras, kafir-caudatums, caudatums, caudatum-bicolors, guinea-kafirs, and durra-caudatums. A similar finding was also reported by Bouchet *et al.* (2012). Another example of clustering by geography was found in accessions from Asia (East, South, and West Asia), which were arbitrarily subdivided into three PGs: PG2, PG3, and PG4. PG3 represented accessions originating from East Asia (China and South Korea) consisting of a mixture of caudatum-bicolors, kafir-bicolors, bicolors, and a caudatum. PG4 comprised accessions from Yemen, including durra-caudatums, guinea-caudatums, and a durra. PG2 consisted primarily of durras from India with the exception of one durra from the countries of Pakistan, Somalia, and Sudan and one kafir from Ethiopia.

The majority of the remaining PGs were formed based on race. The most intriguing were guineas, which were distributed among three PGs. Guineas from West Africa (Cameroon, Nigeria, Ghana, Mali, Benin, Senegal, and Burkina Faso) formed PG6 together with hybrid races also from the region. PG10 contained guinea landraces from the East African countries of Tanzania, Mozambique, and Malawi. The smallest guinea cluster was PG9 with guineas from India. Race caudatum formed two groups, PG7 and PG8. PG7, the major cluster, included caudatums from countries in Africa, Asia, and the United States as well as guinea-caudatums and durra-caudatums. The smaller cluster, PG8, also contained caudatums, guinea-caudatums and durra-caudatums but the accessions in this cluster were solely from Africa. PG1 was a loose cluster of mostly bicolors and caudatum-bicolors. The only group that neither followed race nor geography was PG5, comprised of various races from Africa, the US, Mexico, Syria, and Yemen. Fourteen accessions were not considered clustered (Table S2). Figure 2 illustrates that although kafirs, durras, and guineas each occupied extreme ends of the graph, caudatums were in the middle, closer to the guineas.

### Phylogenetic analysis

**Figure 3:**
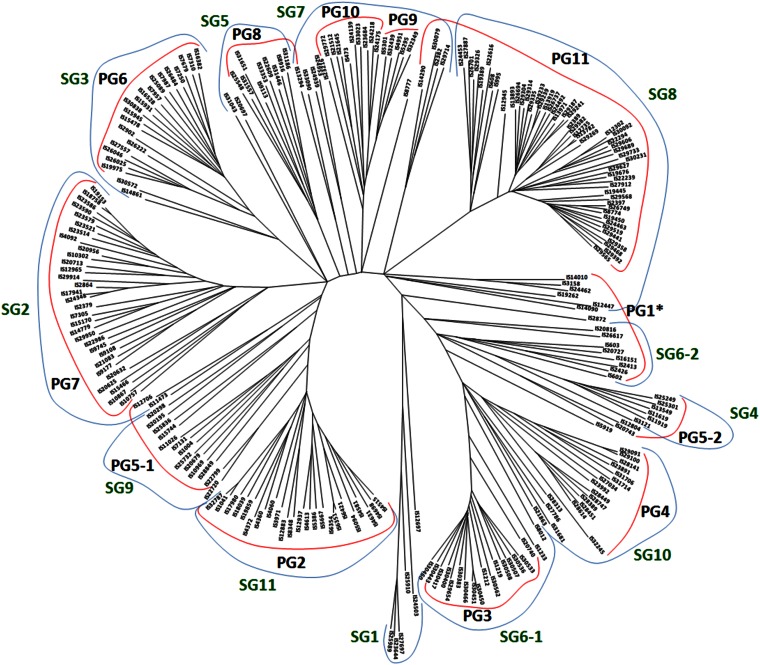
Neighbor-Joining tree of the sorghum mini-core collection based on 13,390 SNP markers. See Figure 2 for corresponding PGs. SG, STRUCTURE group. *IS3158, IS12447, IS14090, IS20816, and IS2413 were not in PG1. See text for details.

Phylogenetic analysis was implemented in MEGA5 using the 13,390 SNP markers (Tamura and Nei 1993). Except for outlying accessions, the clustering pattern was similar to that observed from PCA (Figure 3). The similarity is best illustrated by PG11 containing accessions from Southern Africa (Figure 2). The upper branch in PG11 in Figure 3 (IS14290, IS27887, IS24453, IS26701, and IS19389) corresponds to accessions in the upper part of PG11 in Figure 2. There were some subtle differences between the Neighbor-Joining tree and PCA. For example, PG9, the cluster representing Indian guineas, was more tightly clustered by phylogenetic analysis compared with PCA and it was clustered with PG10 in the Neighbor-Joining tree. Several accessions that were unclustered after PCA (IS3158, IS12447, IS14090, IS20816, and IS2413) formed a loose cluster with accessions from PG1 in the Neighbor-Joining tree. Finally, a discrepancy was observed with PG5 which comprised various races from several countries; it was resolved as two groups in the Neighbor-Joining tree (Figure 3). Despite these differences, the Neighbor-Joining tree showed high congruency with the clusters identified by PCA.

### Population structure

**Figure 4:**
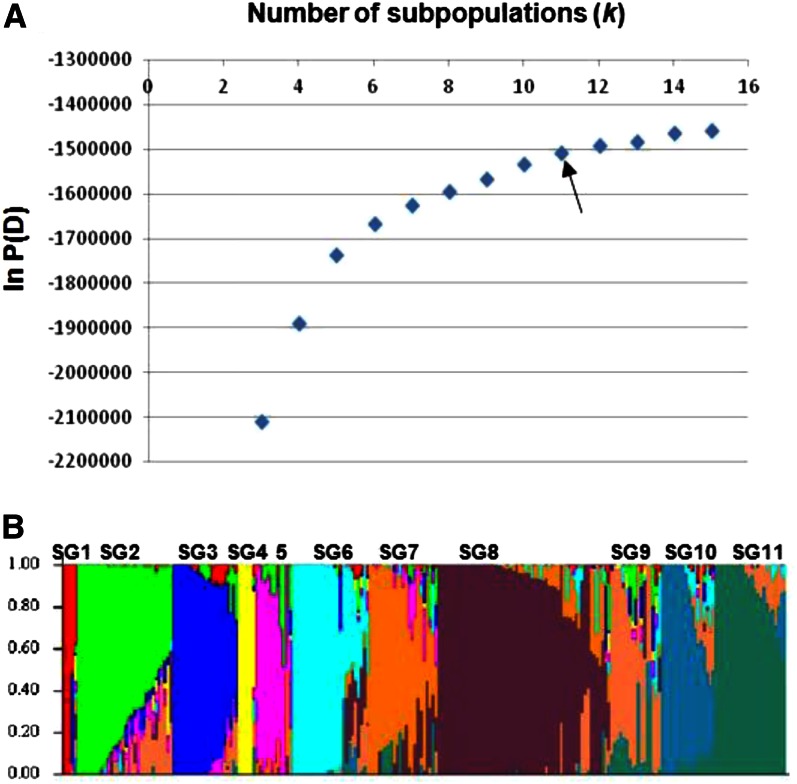
STRUCTURE analysis. (A) Posterior probability, ln *P*(*D*), as a function of the number of subpopulations (*k*). Arrow indicates *k* = 11 used in this study. (B) Population structure for *k* = 11. Clusters are separated by vertical lines with cluster names indicated above the image (SG, STRUCTURE group). Each vertical line in B represents one accession, and the color composition displays the probability of belonging to each of the 11 subpopulations defined by STRUCTURE. See Table S2 for a list of accessions in each SG.

We used the software STRUCTURE to infer the number of possible subpopulations (*k*) in the ICRISAT mini-core collection with the 13,390 SNP markers used for PCA and phylogenetic analysis. To determine the appropriate value of *k*, STRUCTURE was performed 10 times with assumed *k* from 2-16. The *k* value where the posterior probability (ln *P*(*D*)) began to plateau was selected as the true *k* (Casa *et al.* 2008). When *k* was varied from 2 to 16, the posterior probability of the data improved steadily up to *k* = 11–13 and after that it began to plateau (Figure 4A). At *k* = 16, ln *P*(*D*) dropped to less than −2,200,000 (not shown in Figure 4A). Therefore, we compared clustering at *k* = 11, 12, and 13 with those from PCA and phylogenetic analysis to look for the best match. At *k* = 11, STRUCTURE group (SG) 6 included PG3 and half of PG1. When *k* = 12, PG5-1 and PG2 were in one SG, whereas they were separate SGs at *k* = 11. Setting *k* at 13 divided PG7 into two SGs—this is not ideal considering that PG7 was a tight group based on both PCA and phylogenetic analysis (Figures 2 and 3). Based on these results, *k* = 11 (Figure 4B) was chosen to analyze the genetic structure of the mini core collection.

Results from STRUCTURE and from phylogenetic and PCA analyses indicated that 11 subpopulations were more effective in matching clusters of accessions produced by the three methods. Results from STRUCTURE showed that SG7 included two very close neighboring PGs: PGs 9 and 10 containing guineas from India and East Africa, respectively, and all other SGs loosely matched PGs one to one (Figure 3). Comparing the three methods showed that there were a few accessions that clustered differently based on the method of analysis. For example, IS25910, a guinea from the West African country of Ghana and unclustered in the Neighbor-Joining tree, was placed in PG6 by PCA and STRUCTURE; a guinea-durra from the Southern African country of Zimbabwe, IS29733 was grouped in PG8 by PCA but in PG11 by phylogenetic analysis and STRUCTURE (Figure 3 and Table S2). In general, however, results from STRUCTURE were in agreement with those from PCA and phylogenetic analysis. Our results from the three clustering methods demonstrate that bicolor was not sufficiently divergent from the other races to form one inclusive group. However, the other four races were genetically distinct. This is in agreement with conclusions reached in previous studies (Deu *et al.* 1994; Perumal *et al.* 2007; Brown *et al.* 2011). We also found that accessions from Southern Africa formed a tight group, as recently reported (Bouchet *et al.* 2012).

### Analysis of LD

LD analysis was conducted to explore the genome landscape for association studies. The threshold *r*^2^ value (the measure of LD) used in this study was the conventional 0.1 (Remington *et al.* 2001; Nordborg *et al.* 2002; Palaisa *et al.* 2003). Table 1 details the relationship between *r*^2^ and physical distance along all 10 chromosomes.

**Table 1:** Linkage disequilibrium decay as measured by *r*^2^ averaged in distance intervals across the 10 sorghum chromosomes

	SBI-01	SBI-02	SBI-03	SBI-04	SBI-05	SBI-06	SBI-07	SBI-08	SBI-09	SBI-10
<1 kb	0.51	0.37	0.5	0.53	0.43	0.49	0.5	0.44	0.54	0.51
1−10 kb	0.15	0.11	0.12	0.14	0.15	0.14	0.14	0.16	0.22	0.11
10−20 kb	0.1	0.1	0.07	0.11	0.08	0.11	0.11	0.05	0.11	0.1
20−30 kb	0.08	0.15	0.08	0.09	0.07	0.09	0.08	0.1	0.08	0.08
30−50 kb	0.07	0.09	0.08	0.14	0.04	0.1	0.08	0.09	0.08	0.07
50−100 kb	0.07	0.08	0.06	0.07	0.06	0.07	0.06	0.05	0.05	0.05
100 kb−1 Mb	0.04	0.04	0.03	0.04	0.03	0.05	0.04	0.03	0.04	0.03
1−10 Mb	0.03	0.03	0.03	0.03	0.02	0.03	0.03	0.03	0.03	0.03
>10 Mb	0.03	0.03	0.02	0.05	0.04	0.08	0.03	0.06	0.03	0.05
Average	0.05	0.05	0.04	0.05	0.04	0.06	0.05	0.04	0.05	0.05

The average *r*^2^ in each chromosome was similar, between 0.04 and 0.06. If we apply the threshold value of 0.1, the average *r*^2^ falls below 0.1 between 10 and 30 kb depending on the chromosome except for SBI-02 (Table 1). On SBI-02 and -04, there was an increase in *r*^2^ at 20–30 and 30–50 kb, respectively, reflecting LD in this range on these chromosomes. A similar increase was observed on SBI-06 which at >10 Mb had an *r*^2^ value of 0.08, almost identical to the average *r*^2^ values (0.084) at 30–50 kb across all 10 chromosomes. This result was corroborated by the fact that after visually scanning through the chromosome LD plots, no large LD blocks in euchromatic regions were found other than one on SBI-06 (see below). The results agree with Hamblin *et al.* (2005), who found that LD in sorghum largely decays by 10–15 kb. In contrast, Bouchet *et al.* (2012) found a 38% decrease in average *r*^2^ within 50 kb from 0.18 to 0.13.

To demonstrate the difference in LD between euchromatic and heterochromatic regions in the genome, we also compared the average *r*^2^ values between these regions in each chromosome (Table 2). The heterochromatic region in each chromosome was defined according to Mace and Jordan (2011). The average *r*^2^ was greater in euchromatic regions of all sorghum chromosomes except for SBI-06 where a greater average *r*^2^ value was observed in the heterochromatic region.

**Table 2:** Average *r*^2^ value in euchromatic (EC) and heterochromatic (HC) regions in the 10 sorghum chromosomes

	HC Region, Mb	HC	EC
SBI-01	21−50.5	0.048	0.053
SBI-02	17−52	0.043	0.047
SBI-03	17.5−48	0.024	0.045
SBI-04	12.5−43.5	0.057	0.051
SBI-05	17−47.5	0.048	0.041
SBI-06	9.5−39.5	0.099	0.052
SBI-07	10.5−54	0.043	0.054
SBI-08	10−44	0.067	0.039
SBI-09	14−44	0.044	0.049
SBI-10	13−48	0.058	0.045

**Figure 5:**
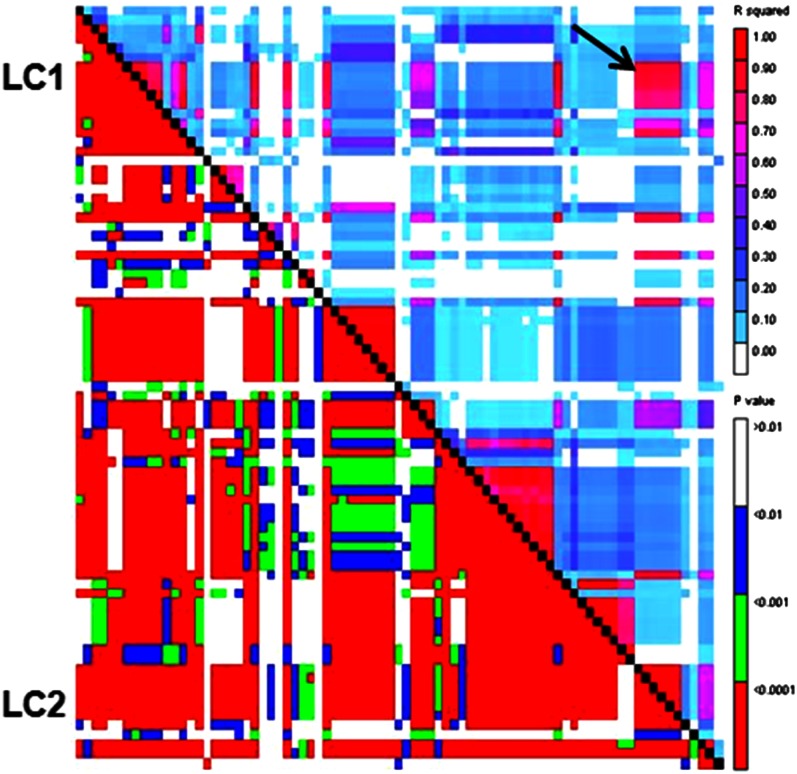
LD in the short arm of sorghum chromosome 6. Arrows indicate the LD block between locus cluster 1 (LC1) and locus cluster 2 (LC2). The scales for *r*^2^ and *p* values are shown to the right of the image. *p* values are shown below the diagonal and *r*^2^ values are shown above.

The sign of large LD blocks on SBI-06 can be seen in Tables 1 and 2. At distances greater than 10 Mb, SBI-06 had an average *r*^2^ value of 0.08, which was 110% greater than the average of other nine chromosomes (Table 1). In addition, the heterochromatic region of SBI-06 had a greater average *r*^2^ value compared with the average *r^2^* value of the heterochromatic regions of the other 9 chromosomes due to the presence of large LD blocks (Table 2). Figure 5 depicts the largest LD block on SBI-06 that starts at six consecutive SNPs (chr6_6718882, chr6_6718899, chr6_6718903, chr6_6960402, chr6_6960403, and chr6_7337793) denoted Locus Cluster 1 (LC1) on the short arm of chromosome 6. LC1 extends through the centromere to LC2, which consists of seven consecutive SNPs (chr6_32273702, chr6_32352964, chr6_32352996, chr6_32352997, chr6_32353005, chr6_32353006, chr6_32353007). The LD block size in this interval averaged 20.33 Mbp and the average *r*^2^ value was 0.85 with an average *p* value of 8.66 × 10^−44^. These results extend the findings of Kim *et al.* (2005) that the euchromatic region on the short arm of SBI-06 has the lowest recombination rate among the 10 chromosomes, 2.3 Mbp/cM compared to an overall average of 0.25 Mbp/cM. Based on cytological characterization, the atypical LD block on the short arm of SBI-06 is probably due to chromosome structure (Kim *et al.* 2005). It should be noted that this large LD block on SBI-06 was detected in individuals from all five races of sorghum. The presence of such a block may hinder efforts to effectively map genes on the short arm of SBI-06.

In addition to the largest LD block spanning the SBI-06 heterochromatic region, two smaller LD blocks were found, one on SBI-06 and the second on SBI-10. The smaller LD block on SBI-06 was 160.79 kb with an average *r*^2^ of 0.66 and a *p* value of 3.46 × 10^−30^. This block was located at ∼52.82 – 52.98 Mbp on the long arm of SBI-06 and was flanked by 6 SNPs on one side and 9 SNPs on the other side (data not shown). The LD block detected on SBI-10 was 4.59 Mb with an *r*^2^ of 1.00 and a *p* value of 3.40 × 10^−5^. It was located between 44.18–48.78 Mbp on SBI-10 and flanked by five SNPs on one side and four on the other (data not shown).

Because LD can be a sign of selection, *i.e.*, selection may create a large LD block that contains domestication-related genes (Takano-Kai *et al.* 2009; Huang *et al.* 2012), we aimed to find smaller LD blocks with equally high *r*^2^ and significant *p* values. Smaller blocks facilitate identification of candidate genes that may have been under selection. We found four such blocks on SBI-01, -02, -09, and -10 and conducted a preliminary examination of the annotated genes in these blocks. Eight genes were found among the 4 LD blocks with four of these having functional annotations as shown in Table 3.

**Table 3:** Linkage disequilibrium blocks and gene content in sorghum chromosomes

	Flanking Single-Nucleotide Polymorphisms					
Chr No.	Left Side	Right Side	Block Size, kb	Average *r*^2^ Value	Average *p* Value	Gene Content
1	chr1_55780969, chr1_55780970, chr1_55780974, chr1_55780977	chr1_55777086, chr1_55777124, chr1_55777130, chr1_55777142	3.8	0.92	0	Sb01g032830
						(*GS3* homolog)
2	chr2_75846165, chr2_75846205, chr2_75846214, chr2_75846217	chr2_75806815, chr2_75806843, chr2_75806854, chr2_75825274, chr2_75825283	20.9-39.7	0.74	1.71 × 10^−42^	Sb02g042100 (4-alpha-glucanotransferase) (two additional genes code for unknown proteins)
9	chr9_57923745, chr9_57947592, chr9_57947624	chr9_57912902, chr9_57912904, chr9_57912941, chr9_57912956	10.8-34.6	0.89	3.72 × 10^−41^	Sb09g029140 (*NF-Y* B subunit) within the 10.8 kb block
10	chr10_3499404	chr10_3463833, chr10_3463834, chr10_3463835, chr10_3463837, chr10_3463838, chr10_3463842, chr10_3463847	35.5	0.77	2.61 × 10^−36^	Sb10g003940 (*FT* homolog) (two additional genes code for unknown proteins)

The block on SBI-01 was one of the strongest LD blocks with an extremely high *r*^2^ value and correspondingly low *p* value. The 3.8-kb block contained just one annotated gene (*SbGS3*; Sb01g032830) homologous to rice *GS3* with 71% identity and 76% similarity based on GenBank BLASTP results (Altschul *et al.* 1997). *GS3* controls grain size and weight in rice (Fan *et al.* 2006) and is shown to be under strong positive selection for the allele conferring larger grain (Takano-Kai *et al.* 2009). LD is increased in rice *GS3* with a 95.7% reduction in nucleotide diversity in accessions carrying the allele which supports larger grain (Takano-Kai *et al.* 2009). These results suggest potential positive selection during sorghum domestication for larger grain leading to LD in the sorghum *GS3* gene. The maize ortholog *ZmGS3* also regulates maize kernel weight and length, but no evidence of selection during maize domestication and improvement is found (Li *et al.* 2010). The LD block on SBI-02 carried a gene encoding 4-α-glucanotransferase, which in potato catalyzes starch breakdown in leaves during the night. Repression of this gene leads to reduced growth in potato (Lloyd *et al.* 2004). The sorghum homolog was mostly expressed in ovary and embryo tissues based on EST profiling (Zeeman *et al.* 2010). Although purely speculative, the present results may indicate a role for the gene in grain development and hence, positive selection leading to LD.

The SBI-10 LD block encoded a homolog to Arabidopsis *Flowering Locus T* (*FT*; 72% identical and 86% similar) and rice *FT* homolog, *Hd3a* (89% identical and 96% similar). *FT* promotes flowering in Arabidopsis in conjunction with *LFY*. Mutations in *FT* delay flowering (Kardailsky *et al.* 1999; Kobayashi *et al.* 1999). *Hd3a* regulates photosensitivity in rice by mediating the transition from vegetative to reproductive growth (Tamaki *et al.* 2007). Recent studies in rice show that several photoperiod genes contain domestication signatures, including high LD within the genes (Huang *et al.* 2012). Like rice, sorghum is a short-day plant, and the LD detected on SBI-10 may reflect selection of the *FT* homolog within this region. Finally, the LD block on SBI-09 harbored a nuclear transcription factor Y subunit B-4 which confers drought tolerance in maize (Nelson *et al.* 2007). Further detailed investigation of the causes of LD that persist in all sorghum races is warranted.

### LD based on race classification

To determine whether LD varies among the races of sorghum, we examined LD after separating each landrace based on its racial classification. The mini-core collection contains the five primary races, thus providing smaller sample sizes for LD analysis. Although it is possible to use small numbers of genotypes to analyze LD in crop plants (Mather *et al.* 2007), this has been shown to result in higher *r*^2^ estimates (Bouchet *et al.* 2012). As expected, separating the landraces into smaller race-specific sets produced higher *r*^2^ estimates (Table 4). The related effect was that the associated *p* values were also greater when compared with estimates using the total population. On average the *r*^2^ increase ranged from 0 to 145% (Table 4).

**Table 4:** Linkage disequilibrium as measured by *r*^2^ and its *p* value in the five primary races of sorghum

Race	No. Accessions	No. Single-Nucleotide Polymorphism Pairs	*r*^2^ > 0.35, %	*p* Values < 0.0001, %	Average *r*^2^	Average *p*
Bicolor	20	511350	7.3	0.8	0.107	0.644
Caudatum	39	583417	6.4	3.2	0.092	0.557
Durra	30	403971	9.5	2.8	0.119	0.572
Guinea	29	451155	10.5	3.6	0.130	0.505
Kafir	21	241203	21.2	1.9	0.226	0.528
All	242	1292724	2.5	18.5	0.092	0.286

We were interested in LD blocks present in one race but absent in others, as these may be a sign that selection (natural or by domestication), random drift, or gene flow has taken place within a given race. Two prominent LD blocks were found on SBI-01 and SBI-02, and in both cases the LD block was specific to race guinea.

**Figure 6:**
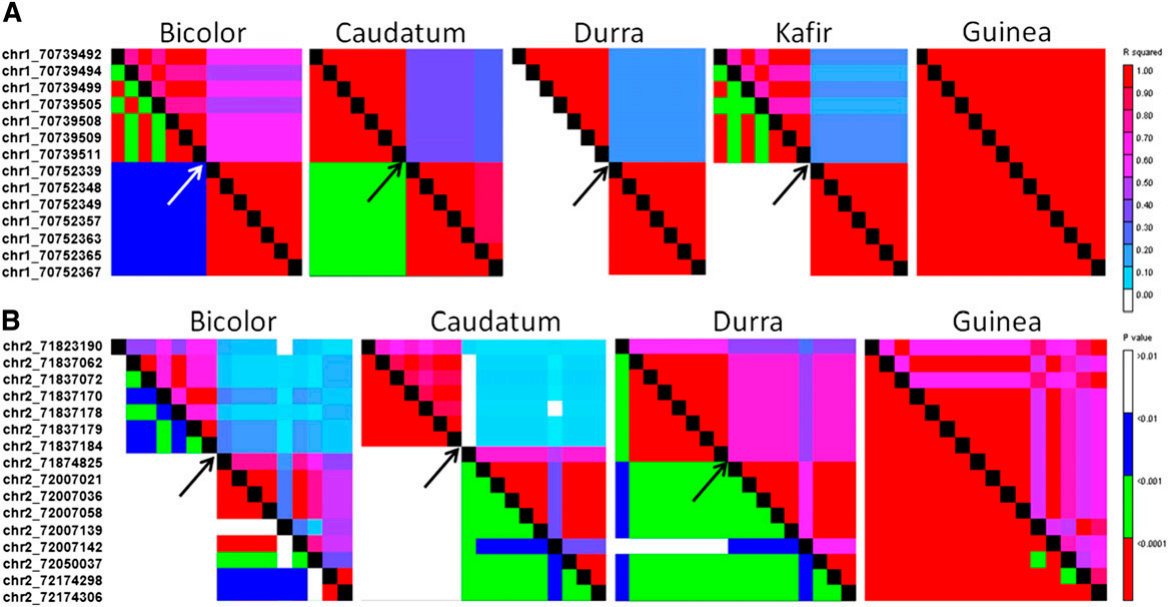
Race-specific linkage disequilibrium blocks in sorghum. (A) SBI-01 LD block specific to race guinea. (B) SBI-02 LD block specific to race guinea. In each plot, *p* values are below the diagonal and *r*^2^ values are above. On the left of each panel are the SNP markers which are also on the X-axis of each plot. On the right is the scale for *r*^2^ and *p* values. Arrows indicate the point of LD breakdown in all races other than guinea. Race kafir is not shown in (B) because of a lack of polymorphic SNPs among accessions within the race in this genomic region.

The LD block on SBI-01 found in guinea was bifurcated between chr1_70739511 and chr1_70752339 in bicolor, caudatum, durra, and kafir (Figure 6A). A search of the sorghum genome identified two genes between 70,739,511 and 70,752,388 bp on SBI-01; gene Sb01g047650 was similar to *Ghd7* and *CONSTANS* in the CCT motif, with the closest rice homolog (Os03g0139500) being 63% identical and 75% similar to Sb01g047650. The other sorghum gene in this genomic region encoded an unknown protein. It is possible that the CCT-motif encoding protein in this region was under selection during domestication of race guinea, but why LD was not detected in the other races is unclear. However, because CCT-domain proteins regulate photoperiodic flowering, vernalization, circadian rhythms, and light signaling, it is possible that an allelic variant of this protein was under selection during the domestication of guineas in Western Africa, although other factors such as genetic drift cannot be excluded.

The second guinea-specific LD block resided on SBI-02 and encoded two genes between 71,837,184 and 71,874,825 bp (Figure 6B): *SbHox14* (Sb02g037560) located between 71,846,508 and 71,847,734 bp and a sugar transporter. *SbHox14* was 66% identical and 75% similar to barley *Vrs1* (*HvHox1*) which is expressed predominantly in the immature spike, the equivalent of a panicle in sorghum (Sakuma *et al.* 2010). *VRS1* changes barley spike morphology by suppressing the lateral spikelets. Mutation in *Vrs1* converts two-rowed barley into six-rowed barley (Komatsuda *et al.* 2007). *SbHox14* was most homologous to the rice gene, Os07g0581700, with 79% identity and 84% similarity based on GenBank BLASTP results (Altschul *et al.* 1997). Os07g0581700 was expressed only in roots and panicles based on GenBank EST profiling. Therefore, *SbHox14* may have a role in regulating panicle architecture in sorghum. Oddly, *SbHox14* is also homologous to *grassy tillers1* (*gt1*) (Whipple *et al.* 2011). *gt1* expression is induced by shading and mutation promotes tillering in maize (Whipple *et al.* 2011). The actual sorghum *gt1* ortholog is Sb01g043910 (*SbGt1*; Whipple *et al.* 2011) which was most homologous to *SbHox14* in the sorghum genome. Further study is needed to determine the function of *SbHox14* in sorghum.

### Conclusions

In this study we analyzed genetic structure and LD in the ICRISAT sorghum mini-core collection that was selected from 22,473 landraces around the world and represents 57 countries. Clustering with 13,390 SNP markers using PCA, phylogenetic and Bayesian analysis of population structure (STRUCTURE) indicated that the collection was structured along both geographic origin and race. Accessions of different races from southern Africa tended to be more similar to each other, as were those from East Asia. Caudatums from different countries were clustered, which was the strongest example of population structured based on race. Guineas from West Africa and durras from India were clustered by race and origin. On the other hand, race bicolor, the most primitive and ancestral of the cultivated races, was found largely distributed among the other four races. This complements reports that sorghum domestication was the product of independent domestication events (Dillon *et al.* 2007; Lin *et al.* 2012).

Genome-wide LD analysis showed that LD decayed within 10−30 kb, close to that previously reported (Hamblin *et al.* 2005). The most prominent LD block was on the short arm of chromosome 6. The block was 20.33 Mb with an *r*^2^ value of 0.85 and a *p* value of 8.66 × 10^−44^, which supports the findings of Kim *et al.* (2005) who first reported that this region on the short arm of SBI-06 had the lowest recombination rate among all sorghum chromosomes. Smaller LD blocks also were detected, which may reflect selection during the domestication of sorghums. An LD block on SBI-01contained a *GS3* homolog and the SBI-10 block contained an *FT* homolog. *GS3* determines grain size in rice and *Hd3a*, the rice homolog of *FT*, affects photosensitivity. Both *GS3* and *Hd3a* have been shown to be targets of selection during rice domestication. The fact that both of these genes are located in a region with increased LD suggests that they may have been subject to selection in sorghum as well. Race-specific LD blocks were found only in race guinea, presumably the most recent of the cultivated races. Two genes were identified in these blocks: a CCT domain-containing protein and a homeobox gene (*SbHox14*). *SbHox14* is similar to barley *Vrs1*, in which a mutation converts two-rowed barley into six-rowed barley, again affecting panicle (spike) morphology. Although purely correlative, these smaller LD regions encode genes related to photosensitivity and panicle/grain morphology. As the sorghum races were established on panicle architecture and geographical origin, the relationship between circadian clock-associated and panicle morphology genes and regions of LD across the sorghum genome warrant further investigation.

## Supplementary Material

Supporting Information

## References

[bib1] Altschul S. F. Madden T. L. Schäffer A. A. Zhang J. Zhang Z., 1997 Gapped BLAST and PSI-BLAST: a new generation of protein database search programs. Nucleic Acids Res. 25: 3389–3402 9254694 10.1093/nar/25.17.3389 PMC146917

[bib2] Baird N. A. Etter P. D. Atwood T. S. Currey M. C. Shiver A. L., 2008 Rapid SNP discovery and genetic mapping using sequenced RAD markers. PLoS ONE 3: e3376 18852878 10.1371/journal.pone.0003376 PMC2557064

[bib3] Becker R. A Chambers J. M. Wilks A. R., 1988 The New S Language. Wadsworth & Brooks/Cole, Pacific Grove, PA

[bib4] Bergelson J. Roux F., 2010 Towards identifying genes underlying ecologically relevant traits in *Arabidopsis thaliana*. Nat. Rev. Genet. 11: 867–879 21085205 10.1038/nrg2896

[bib5] Bouchet S. Pot D. Deu M. Rami J.-F. Billot C., 2012 Genetic structure, linkage disequilibrium and signature of selection in sorghum: lessons from physically anchored DArT markers. PLoS ONE 7: e33470 22428056 10.1371/journal.pone.0033470 PMC3302775

[bib6] Bradbury P. J. Zhang Z. Kroon D. E. Casstevens T. M. Ramdoss Y., 2007 TASSEL: Software for association mapping of complex traits in diverse samples. Bioinformatics 23: 2633–2635 17586829 10.1093/bioinformatics/btm308

[bib7] Brown P. J. Myles S. Kresovich S., 2011 Genetic support for phenotype-based racial classification in sorghum. Crop Sci. 51: 224–230

[bib8] Casa A. M. Mitchell S. E. Hamblin M. T. Sun H. Bowers J. E., 2005 Diversity and selection in sorghum: Simultaneous analyses using simple sequence repeats. Theor. Appl. Genet. 111: 23–30 15864526 10.1007/s00122-005-1952-5

[bib9] Casa A. Pressoir G. Brown P. Mitchell S. Rooney W., 2008 Community resources and strategies for association mapping in sorghum. Crop Sci. 48: 30–40

[bib10] Cui Y. X. Xu G. W. Magill C. W. Schertz K. F. Hart G. E., 1995 RFLP-based assay of *Sorghum bicolor* (L.) Moench genetic diversity. Theor. Appl. Genet. 90: 787–796 24172920 10.1007/BF00222013

[bib11] Dahlberg J. A. Zhang X. Hart G. E. Mullet J. E., 2002 Comparative assessment of variation among sorghum germplasm accessions using seed morphology and RAPD measurements. Crop Sci. 42: 291–296 11756288 10.2135/cropsci2002.2910

[bib12] Davey J. W. Blaxter M. L., 2010 RADSeq: Next-generation population genetics. Brief. Funct. Genomics 9: 416–423 21266344 10.1093/bfgp/elq031 PMC3080771

[bib13] de Oliveira A. C. Richter T. Bennetzen J. L., 1996 Regional and racial specificities in sorghum germplasm assessed with DNA markers. Genome 39: 579–587 8675002 10.1139/g96-073

[bib14] de Wet J. M. J., 1978 Systematics and evolution of sorghum sect. Sorghum (Gramineae). Am. J. Bot. 65: 477–484

[bib15] de Wet J. M. J. Huckabay J. P., 1967 The origin of *Sorghum bicolor*. II. Distribution and domestication. Evolution 21: 787–802 10.1111/j.1558-5646.1967.tb03434.x 28563076

[bib16] Deu M. Gonzalez-de-Leon D. Glaszmann J. C. Degremont I. Chantereau J., 1994 RFLP diversity in cultivated sorghum in relation to racial differentiation. Theor. Appl. Genet. 88: 838–844 24186186 10.1007/BF01253994

[bib17] Deu M. Sagnard F. Chantereau J. Calatayud C. Hérault D., 2008 Niger-wide assessment of *in situ* sorghum genetic diversity with microsatellite markers. Theor. Appl. Genet. 116: 903–913 18273600 10.1007/s00122-008-0721-7

[bib18] Dillon S. L. Shapter F. M. Henry R. J. Cordeiro G. Izquierdo L., 2007 Domestication to crop improvement: Genetic resources for *Sorghum* and *Saccharum* (Andropogoneae). Ann. Bot. (Lond.) 100: 975–989 10.1093/aob/mcm192 PMC2759214 17766842

[bib19] Djè Y. Heuertz M. Lefèbvre C. Vekemans X., 2000 Assessment of genetic diversity within and among germplasm accessions in cultivated sorghum using microsatellite markers. Theor. Appl. Genet. 100: 918–925

[bib20] Elshire R. J. Glaubitz J. C. Sun Q. Poland J. A. Kawamoto K., 2011 A robust, simple genotyping-by-sequencing (GBS) approach for high diversity species. PLoS ONE 6: e19379 21573248 10.1371/journal.pone.0019379 PMC3087801

[bib21] Fan C. Xing Y. Mao H. Lu T. Han B., 2006 *GS3*, a major QTL for grain length and weight and minor QTL for grain width and thickness in rice, encodes a putative transmembrane protein. Theor. Appl. Genet. 112: 1164–1171 16453132 10.1007/s00122-006-0218-1

[bib22] Folkertsma R. T. Rattunde H. F. W. Chandra S. Raju G. S. Hash C. T., 2005 The pattern of genetic diversity of guinea-race *Sorghum bicolor* (L.) Moench landraces as revealed with SSR markers. Theor. Appl. Genet. 111: 399–409 15965652 10.1007/s00122-005-1949-0

[bib23] Ghebru B. G. Schmidt R. S. Bennetzen J. B., 2002 Genetic diversity of Eritrean sorghum landraces assessed with simple sequence repeat (SSR) markers. Theor. Appl. Genet. 105: 229–236 12582524 10.1007/s00122-002-0929-x

[bib24] Gore M. A. Chia J.-M. Elshire R. J. Sun Q. Ersoz E. S., 2009 A first-generation haplotype map of maize. Science 326: 1115–1117 19965431 10.1126/science.1177837

[bib25] Grenier C. Hamon P. Bramel-Cox P. J., 2001 Core collection of sorghum: II. Comparison of three random sampling strategies. Crop Sci. 41: 241–246

[bib26] Gupta P. Rustgi S. Kulwal P., 2005 Linkage disequilibrium and association studies in higher plants: Present status and future prospects. Plant Mol. Biol. 57: 461–485 15821975 10.1007/s11103-005-0257-z

[bib27] Hamblin M. T. Salas Fernandez M. G. Casa A. M. Mitchell S. E. Paterson A. H., 2005 Equilibrium processes cannot explain high levels of short- and medium-range linkage disequilibrium in the domesticated grass *Sorghum bicolor*. Genetics 171: 1247–1256 16157678 10.1534/genetics.105.041566 PMC1456844

[bib28] Harlan J. R. de Wet J. M. J., 1972 A simplified classification of cultivated sorghum. Crop Sci. 12: 172–176

[bib29] Huang C.-L. Hung C.-Y. Chiang Y.-C. Hwang C.-C. Hsu T.-W., 2012 Footprints of natural and artificial selection for photoperiod pathway genes in *Oryza*. Plant J. 70: 769–782 22268451 10.1111/j.1365-313X.2012.04915.x

[bib30] Kardailsky I. Shukla V. K. Ahn J. H. Dagenais N. Christensen S. K., 1999 Activation tagging of the floral inducer *FT*. Science 286: 1962–1965 10583961 10.1126/science.286.5446.1962

[bib31] Kim J.-S. Islam-Faridi M. N. Klein P. E. Stelly D. M. Price H. J., 2005 Comprehensive molecular cytogenetic analysis of sorghum genome architecture: Distribution of euchromatin, heterochromatin, genes and recombination in comparison to rice. Genetics 171: 1963–1976 16143604 10.1534/genetics.105.048215 PMC1456119

[bib32] Kimber C. T., 2000 Origins of domesticated sorghum and its early diffusion to India and China, pp. 3–98 in Sorghum, edited by Smith C. W. Frederiksen R. A. John Wiley, New York

[bib33] Kobayashi Y. Kaya H. Goto K. Iwabuchi M. Araki T., 1999 A pair of related genes with antagonistic roles in mediating flowering signals. Science 286: 1960–1962 10583960 10.1126/science.286.5446.1960

[bib34] Komatsuda T. Pourkheirandish M. He C. Azhaguvel P. Kanamori H., 2007 Six-rowed barley originated from a mutation in a homeodomain-leucine zipper I-class homeobox gene. Proc. Natl. Acad. Sci. USA 104: 1424–1429 17220272 10.1073/pnas.0608580104 PMC1783110

[bib35] Li Q. Yang X. Bai G. Warburton M. Mahuku G., 2010 Cloning and characterization of a putative *GS3* ortholog involved in maize kernel development. Theor. Appl. Genet. 120: 753–763 19898828 10.1007/s00122-009-1196-x

[bib36] Lin Z. Li X. Shannon L. M. Yeh C.-T. Wang M. L., 2012 Parallel domestication of the *shattering1* genes in cereals. Nat. Genet. 44: 720–724 22581231 10.1038/ng.2281 PMC3532051

[bib37] Lloyd J. R. Blennow A. Burhenne K. Kossmann J., 2004 Repression of a novel isoform of disproportionating enzyme (stdpe_2_) in potato leads to inhibition of starch degradation in leaves but not tubers stored at low temperature. Plant Physiol. 134: 1347–1354 15034166 10.1104/pp.103.038026 PMC419812

[bib38] Mace E. Jordan D., 2011 Integrating sorghum whole genome sequence information with a compendium of sorghum QTL studies reveals uneven distribution of QTL and of gene-rich regions with significant implications for crop improvement. Theor. Appl. Genet. 123: 169–191 21484332 10.1007/s00122-011-1575-y

[bib39] Mace E. S. Xia L. Jordan D. R. Halloran K. Parh D. K., 2008 DArT markers: Diversity analyses and mapping in *Sorghum bicolor*. BMC Genomics 9: 10.1186/1471-2164-1189-1126 PMC2270266 18208620

[bib40] Mann J. A. Kimber C. T. Miller F. R., 1983 The origin and early cultivation of sorghums in Africa. Bull B Texas Agric Exper Station 1454: 107–153

[bib41] Mather K. A. Caicedo A. L. Polato N. R. Olsen K. M. McCouch S., 2007 The extent of linkage disequilibrium in rice (*Oryza sativa* L.). Genetics 177: 2223–2232 17947413 10.1534/genetics.107.079616 PMC2219496

[bib42] Menkir A. Goldsbrough P. Ejeta G., 1997 RAPD based assessment of genetic diversity in cultivated races of sorghum. Crop Sci. 37: 564–569

[bib43] Menz M. A. Klein R. R. Unruh N. C. Rooney W. L. Klein P. E., 2004 Genetic diversity of public inbreds of sorghum determined by mapped AFLP and SSR markers. Crop Sci. 44: 1236–1244

[bib44] Nelson D. E. Repetti P. P. Adams T. R. Creelman R. A. Wu J., 2007 Plant nuclear factor Y (NF-Y) B subunits confer drought tolerance and lead to improved corn yields on water-limited acres. Proc. Natl. Acad. Sci. USA 104: 16450–16455 17923671 10.1073/pnas.0707193104 PMC2034233

[bib45] Nelson J. Wang S. Wu Y. Li X. Antony G., 2011 Single-nucleotide polymorphism discovery by high-throughput sequencing in sorghum. BMC Genomics 12: 352 21736744 10.1186/1471-2164-12-352 PMC3146956

[bib46] Nordborg M. Borevitz J. Bergelson J. Berry C. Chory J., 2002 The extent of linkage disequilibrium in *Arabidopsis thaliana*. Nat. Genet. 30: 190–193 11780140 10.1038/ng813

[bib47] Palaisa K. A. Morgante M. Williams M. Rafalski A., 2003 Contrasting effects of selection on sequence diversity and linkage disequilibrium at two phytoene synthase loci. Plant Cell 15: 1795–1806 12897253 10.1105/tpc.012526 PMC167170

[bib48] Paterson A. H. Bowers J. E. Bruggmann R. Dubchak I. Grimwood J., 2009 The *Sorghum bicolor* genome and the diversification of grasses. Nature 457: 551–556 19189423 10.1038/nature07723

[bib49] Perumal R. Krishnaramanujam R. Menz M. A. Katilé S. Dahlberg J., 2007 Genetic diversity among sorghum races and working groups based on AFLPs and SSRs. Crop Sci. 47: 1375–1383

[bib50] Pritchard J. K. Stephens M. Donnelly P., 2000 Inference of population structure using multilocus genotype data. Genetics 155: 945–959 10835412 10.1093/genetics/155.2.945 PMC1461096

[bib51] Remington D. L. Thornsberry J. M. Matsuoka Y. Wilson L. M. Whitt S. R., 2001 Structure of linkage disequilibrium and phenotypic associations in the maize genome. Proc. Natl. Acad. Sci. USA 98: 11479–11484 11562485 10.1073/pnas.201394398 PMC58755

[bib52] Ripley B. D., 2001 The R project in statistical computing. MSOR Connections. The newsletter of the LTSN Maths. Stats & OR Network 1: 23–25

[bib53] Ritter K. Mcintyre C. Godwin I. Jordan D. Chapman S., 2007 An assessment of the genetic relationship between sweet and grain sorghums, within *Sorghum bicolor* ssp. *bicolor* (L.) Moench, using AFLP markers. Euphytica 157: 161–176

[bib54] Sakuma S. Pourkheirandish M. Matsumoto T. Koba T. Komatsuda T., 2010 Duplication of a well-conserved homeodomain-leucine zipper transcription factor gene in barley generates a copy with more specific functions. Funct. Integr. Genomics 10: 123–133 19707806 10.1007/s10142-009-0134-y PMC2834773

[bib55] Scheet P. Stephens M., 2006 A fast and flexible statistical model for large-scale population genotype data: Applications to inferring missing genotypes and haplotypic phase. Am. J. Hum. Genet. 78: 629–644 16532393 10.1086/502802 PMC1424677

[bib56] Smith C. W., 1995 *Crop Production: Evolution*, *History*, *and Technology*. John Wiley & Sons, New York

[bib57] Stemler A. B. L. Harlan J. R. Dewet J. M. J., 1975 Caudatum sorghums and speakers of Chari-Nile languages in Africa. J. Afr. Hist. 16: 161–183

[bib58] Takano-Kai N. Jiang H. Kubo T. Sweeney M. Matsumoto T., 2009 Evolutionary history of *GS3*, a gene conferring grain length in rice. Genetics 182: 1323–1334 19506305 10.1534/genetics.109.103002 PMC2728869

[bib59] Tamaki S. Matsuo S. Wong H. L. Yokoi S. Shimamoto K., 2007 Hd3a protein is a mobile flowering signal in rice. Science 316: 1033–1036 17446351 10.1126/science.1141753

[bib60] Tamura K. Nei M., 1993 Estimation of the number of nucleotide substitutions in the control region of mitochondrial DNA in humans and chimpanzees. Mol. Biol. Evol. 10: 512–526 8336541 10.1093/oxfordjournals.molbev.a040023

[bib61] Tamura K. Peterson D. Peterson N. Stecher G. Nei M., 2011 Mega5: Molecular evolutionary genetics analysis using maximum likelihood, evolutionary distance, and maximum parsimony methods. Mol. Biol. Evol. 28: 2731–2739 21546353 10.1093/molbev/msr121 PMC3203626

[bib62] Upadhyaya H. Wang Y.-H. Sharma S. Singh S. Hasenstein K., 2012 SSR markers linked to kernel weight and tiller number in sorghum identified by association mapping. Euphytica 187: 401–410

[bib63] Upadhyaya H. D. Pundir R. P. S. Dwivedi S. L. Gowda C. L. L. Reddy V. G., 2009 Developing a mini core collection of sorghum for diversified utilization of germplasm. Crop Sci. 49: 1769–1780

[bib64] Wang Y.-H. Poudel D. D. Hasenstein K. H., 2011 Identification of SSR markers associated with saccharification yield using pool-based genome-wide association mapping in sorghum. Genome 54: 883–889 21999235 10.1139/g11-055

[bib65] Wang Y.-H. Bible P. Loganantharaj R. Upadhyaya H., 2012 Identification of SSR markers associated with height using pool-based genome-wide association mapping in sorghum. Mol. Breed. 30: 281–292

[bib66] Whipple C. J. Kebrom T. H. Weber A. L. Yang F. Hall D., 2011 *Grassy tillers1* promotes apical dominance in maize and responds to shade signals in the grasses. Proc. Natl. Acad. Sci. USA 108: E506–E512 21808030 10.1073/pnas.1102819108 PMC3158142

[bib67] Zeeman S. C. Kossmann J. Smith A. M., 2010 Starch: Its metabolism, evolution, and biotechnological modification in plants. Annu. Rev. Plant Biol. 61: 209–234 20192737 10.1146/annurev-arplant-042809-112301

