# Supplementary material for: Genetic Structure and Linkage Disequilibrium in a Diverse, Representative Collection of the C4 Model Plant, Sorghum bicolor
Source: G3 (Bethesda). 2013 May 1;3(5):783–93. doi: 10.1534/g3.112.004861 (PMC3656726; doi:10.1534/g3.112.004861)
Supplement: Supporting Information [file supp_3_5_783__index.html]

Supporting Information 

# Genetic Structure and Linkage Disequilibrium in a Diverse, Representative Collection of the C4 Model Plant, *Sorghum bicolor*

## Supporting Information for Wang *et al.*, 2013

**Files in this Data Supplement:**

- Supporting Information - Figure S1 and Tables S1-S2 (PDF, 351 KB)
- Figure S1 - a) Distribution of SNPs utilized in this study across the 10 *Sorghum bicolor* chromosomes. b) Distance between SNPs utilized in this study. (PDF, 198 KB)
- Table S2 - Clustering of the sorghum mini core accessions by principal component analysis and STRUCTURE using 13,390 SNP markers. (PDF, 190 KB)
- Table S1 - Genotype data for 242 accession mini core collection and BTx623 reference genotype following genotyping-by-sequencing with *FseI*-digested DNA and imputation of missing data with fastPHASE. (.zip, 47.4 MB)
