# Supplementary material for: Genetic Structure and Linkage Disequilibrium in a Diverse, Representative Collection of the C4 Model Plant, Sorghum bicolor
Source: G3 (Bethesda). 2013 May 1;3(5):783–93. doi: 10.1534/g3.112.004861 (PMC3656726; doi:10.1534/g3.112.004861)
Supplement: Supporting Information [file supp_3.5.783_004861SI.pdf]

G3 · Genes | Genomes | Genetics

Genetic structure and linkage disequilibrium in a diverse, representative collection of the C4 model plant, *Sorghum bicolor*.

Yi-Hong Wang[*,¥], Hari D. Upadhyaya[§,¥], A. Millie Burrell[†], Sayed Mohammad Ebrahim Sahraeian[‡], Robert R. Klein[**], Patricia E. Klein[†,1]

[¥] Contributed equally to this work.

[*]Department of Biology, University of Louisiana at Lafayette, Lafayette, LA 70504, USA.
[§]Gene Bank, International Crops Research Institute for the Semi-Arid Tropics (ICRISAT), Patancheru 502 324, Andhra Pradesh, India.
[†]Department of Horticultural Sciences and Institute for Plant Genomics and Biotechnology, Texas A&M University, College Station, TX 77843, USA.
[‡]Department of Electrical and Computer Engineering, Texas A&M University, College Station, TX 77843, USA.
[**]USDA-ARS, Southern Plains Agricultural Research Center, College Station, TX 77845, USA.

[1]Corresponding author:

Patricia E. Klein
Department of Horticultural Sciences and Institute for Plant Genomics and Biotechnology
Texas A&M University
TAMU 2123
College Station, TX 77843
pklein@tamu.edu
phone: 979-862-6308

DOI: 10.1534/g3.112.004861

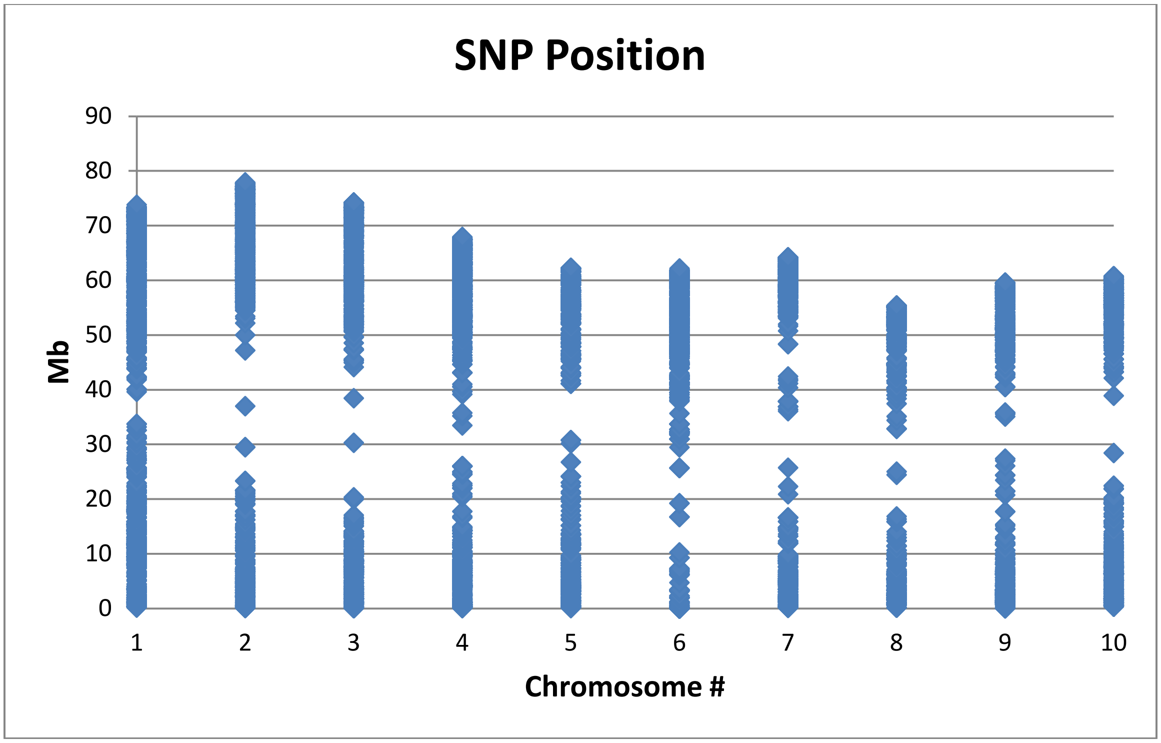

Figure S1a Distribution of SNPs utilized in this study across the 10 *Sorghum bicolor* chromosomes. The location of SNPs generated by genotyping-by-sequencing at *FseI* cut-sites are represented by blue squares. The y-axis represents the physical location of the SNPs across the sorghum genome. The SNPs are well-distributed over the 10 chromosomes and cover predominantly euchromatic regions of the genome. Regions in which SNPs are absent represent the gene-poor pericentromeric heterochromatic regions.

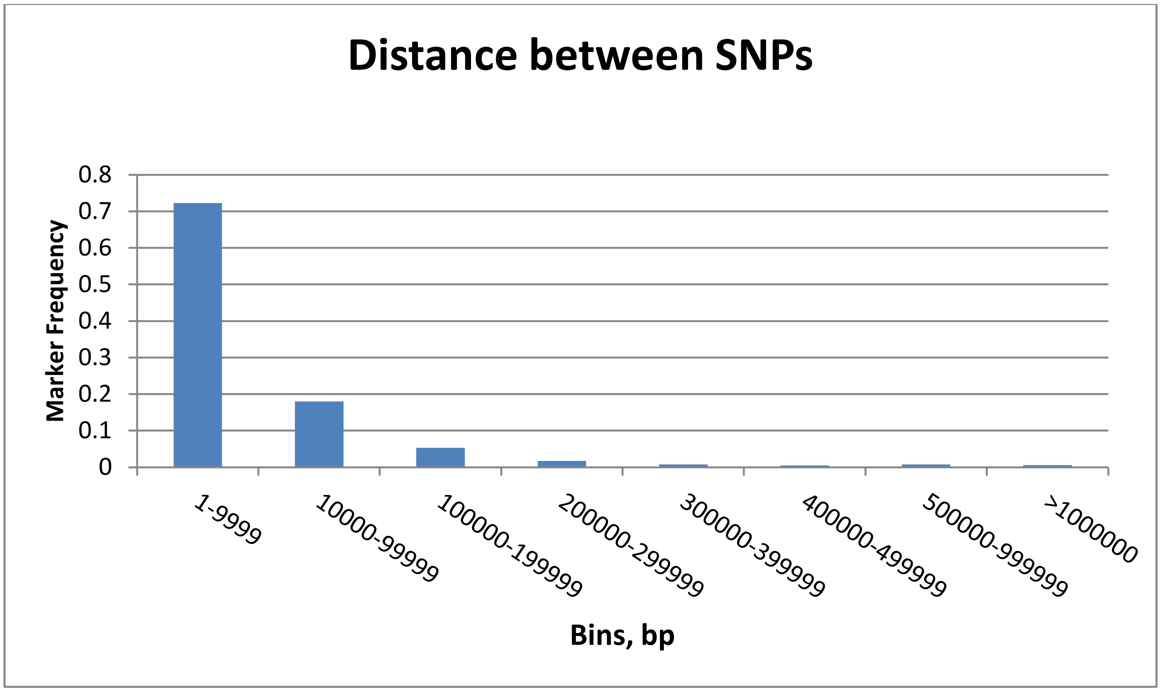

Figure S1b Distance between SNPs utilized in this study. The x-axis represents ranges of base pair distances between adjacent SNPs. The y-axis represents the percentage of total SNPs that fall within a specific range. The figure shows that 72% of the SNPs fall within 10,000 base pairs of each other.

Table S1 is available for download at http://www.g3journal.org/lookup/suppl/doi:10.1534/g3.112.004861/-/DC1.

Table S1 Genotype data for 242 accession mini core collection and BTx623 reference genotype following genotyping-by-sequencing with *Fse*I-digested DNA and imputation of missing data with fastPHASE. SNPs were detected using the CLC Genomics Workbench SNP detection tool following mapping of sequences from each line to the BTx623 reference genome.

Table S2 Clustering of the sorghum mini core accessions by principal component analysis and STRUCTURE using 13,390 SNP markers.

PCA groups	Accession	Race	Country		SGs	Accession	Race	Country
PG1	IS602	B	USA		SG1	23644	G	Gambia
	IS603	B	USA			25989	G	Mali
	IS608	B	USA			27697	G	SierraLeone
	IS2426	CB	Afghan			25910	G	Mali
	IS2872	CB	Egypt			24503	B	S Africa
	IS14010	CB	S Africa		SG2	10302	C	Thailand
	IS19262	GC	Sudan			19153	GC	Sudan
	IS20727	B	USA			23521	GC	Ethiopia
	IS21863	B	Syrian			23579	GC	Ethiopia
	IS22616	B	Myanmar			23586	GC	Ethiopia
	IS24462	CB	S Africa			23590	GC	Ethiopia
	IS26617	CB	Madagascar			24348	C	India
	IS27786	DB	Morocco			23514	C	Ethiopia
	IS31681	B	Algeria			17941	C	India
PG2	IS1041	D	India			4092	C	India
	IS3971	D	India			2864	C	S Africa
	IS4060	DB	India			20713	GC	USA
	IS4360	D	India			12965	C	Cuba
	IS4372	GD	India			29914	C	Zimbabwe
	IS4515	D	India			20956	DC	Indonesia
	IS4581	D	India			2379	C	S Africa
	IS4613	D	India			21083	C	Kenya
	IS4631	D	India			15170	C	Cameroon
	IS4698	D	India			29950	GC	Zimbabwe
	IS5094	D	India			9177	C	Kenya
	IS5386	D	India			7305	C	Nigeria
	IS5667	D	India			14779	C	Cameroon
	IS5919	D	India			9745	C	Sudan
	IS6351	D	India			22986	C	Sudan
	IS6354	D	India			20632	C	USA
	IS6421	D	India			20697	C	USA
	IS8348	D	Pakistan			10757	C	Chad
	IS12883	D	India			11473	C	Ethiopia
	IS12937	K	Ethiopia			15466	C	Cameroon
	IS17980	D	India			20625	DC	USA
	IS18039	DB	India			14090	C	Argentina
	IS19859	D	India		SG3	7250	G	Nigeria
	IS22799	D	Somalia			7310	G	Nigeria

	IS32787	D	Somalia			7679	G	Nigeria
PG3	IS1212	KB	China			15931	G	Cameroon
	IS1219	GB	China			16382	G	Cameroon
	IS20740	B	USA			26484	G	Benin
	IS29654	KB	China			7957	GB	Nigeria
	IS30383	CB	China			7987	G	Nigeria
	IS30400	CB	China			25089	G	Ghana
	IS30417	CB	China			15478	GC	Cameroon
	IS30443	CB	China			15945	GC	Cameroon
	IS30450	CB	China			16528	G	Cameroon
	IS30451	CB	China			30838	G	Cameroon
	IS30460	C	China			26046	G	Mali
	IS30466	CB	China			26025	G	Mali
	IS30507	CB	Korea			19975	G	Senegal
	IS30508	CB	Korea			27557	G	BurkinaFaso
	IS30533	CB	Korea			2902	CB	Nigeria
	IS30536	CB	Korea			26222	GC	Togo
	IS30562	B	Korea			10867	GC	Chad
PG4	IS22720	D	Somalia			14861	C	Cameroon
	IS23891	D	Yemen			30572	GC	Cameroon
	IS23992	C	Yemen		SG4	11619	DB	Ethiopia
	IS27034	D	Sudan			11919	DB	Ethiopia
	IS28141	DC	Yemen			13549	CB	Mexico
	IS28389	DC	Yemen			25249	DB	Ethiopia
	IS28449	GC	Yemen			25301	DB	Ethiopia
	IS28451	GC	Yemen			3121	B	USA
	IS28614	DC	Yemen		SG5	25548	C	Rwanda
	IS28747	DC	Yemen			33353	C	Kenya
	IS29091	DC	Yemen			22609	C	Sri Lanka
	IS29100	DC	Yemen			31557	C	Burundi
	IS31706	D	Yemen			31446	GC	Uganda
	IS31714	DC	Yemen			9113	C	Kenya
	IS32245	DC	Yemen			31186	GC	Uganda
PG 5	IS1004	D	India			31651	C	Zaire
	IS3121	B	USA			8916	GC	Uganda
	IS7131	DC	Uganda			9108	C	Kenya
	IS8012	B	Japan			24939	B	Zambia
	IS10969	GC	USA			31043	C	Uganda
	IS11026	D	Ethiopia		SG6	1212	KB	China
	IS11619	DB	Ethiopia			29654	KB	China
	IS11919	DB	Ethiopia			30383	CB	China

	IS12706	CB	USA			30400	CB	China
	IS12804	B	Turkey			30417	CB	China
	IS13549	CB	Mexico			30443	CB	China
	IS15744	DC	Cameroon			30450	CB	China
	IS16151	CB	Cameroon			30451	CB	China
	IS20195	B	Niger			30460	C	China
	IS20679	GC	USA			30466	CB	China
	IS20743	B	USA			30507	CB	Korea
	IS25249	DB	Ethiopia			30508	CB	Korea
	IS25301	DB	Ethiopia			30533	CB	Korea
	IS25732	D	Mali			30536	CB	Korea
	IS25836	D	Mali			30562	B	Korea
	IS28313	DC	Yemen			1219	GB	China
	IS28849	DC	Yemen			1233	B	China
PG6	IS2902	CB	Nigeria			20740	B	USA
	IS7250	G	Nigeria			16151	CB	Cameroon
	IS7310	G	Nigeria			603	B	USA
	IS7679	G	Nigeria			20727	B	USA
	IS7957	GB	Nigeria			8012	B	Japan
	IS7987	G	Nigeria			20816	B	USA
	IS10867	GC	Chad			602	B	USA
	IS15478	GC	Cameroon			2426	CB	Afghan
	IS15931	G	Cameroon			20743	B	USA
	IS15945	GC	Cameroon		SG7	23684	G	Mozambique
	IS16382	G	Cameroon			24218	G	Tanzania
	IS16528	G	Cameroon			24175	G	Tanzania
	IS19975	G	Senegal			21645	G	Malawi
	IS20956	DC	Indonesia			5295	G	India
	IS25089	G	Ghana			5301	GC	India
	IS25910	G	Mali			32349	G	India
	IS26025	G	Mali			4951	G	India
	IS26046	G	Mali			24139	G	Tanzania
	IS26222	GC	Togo			473	GK	USA
	IS26484	G	Benin			32439	G	India
	IS27557	G	BurkinaFaso			29772	GC	Zimbabwe
	IS30838	G	Cameroon			21512	G	Malawi
PG7	IS2379	C	S Africa			33023	G	Tanzania
	IS2864	C	S Africa			24953	GC	Zambia
	IS4092	C	India			23216	CB	Zambia
	IS7305	C	Nigeria			2382	C	S Africa
	IS9108	C	Kenya			30079	DC	Zimbabwe

	IS9177	C	Kenya			13294	CB	Venezuela
	IS9745	C	Sudan			29714	KD	Zimbabwe
	IS10302	C	Thailand			8777	CB	Uganda
	IS10757	C	Chad			26617	CB	Madagascar
	IS11473	C	Ethiopia			33090	D	Honduras
	IS12965	C	Cuba		SG8	2389	K	S Africa
	IS14779	C	Cameroon			2397	K	S Africa
	IS14861	C	Cameroon			8774	KD	S Africa
	IS15170	C	Cameroon			12735	CB	Saudi
	IS15466	C	Cameroon			13782	KD	S Africa
	IS17941	C	India			13919	KC	S Africa
	IS19153	GC	Sudan			13971	C	S Africa
	IS20298	CB	Niger			19450	GK	Botswana
	IS20625	DC	USA			24463	K	S Africa
	IS20632	C	USA			24492	K	S Africa
	IS20713	GC	USA			26737	K	S Africa
	IS21083	C	Kenya			26749	K	S Africa
	IS22986	C	Sudan			29233	K	Swaziland
	IS23514	C	Ethiopia			29239	K	Swaziland
	IS23521	GC	Ethiopia			29241	KC	Swaziland
	IS23579	GC	Ethiopia			29304	GK	Swaziland
	IS23586	GC	Ethiopia			29519	KC	Lesotho
	IS23590	GC	Ethiopia			29314	DC	Swaziland
	IS23644	G	Gambia			29565	GC	Lesotho
	IS24348	C	India			29582	K	Lesotho
	IS25989	G	Mali			19445	K	Botswana
	IS27697	G	SierraLeone			29392	K	Lesotho
	IS29914	C	Zimbabwe			13893	KC	S Africa
	IS29950	GC	Zimbabwe			29441	KC	Lesotho
	IS31043	C	Uganda			29269	GC	Swaziland
	IS8916	GC	Uganda			26694	C	S Africa
PG 8	IS9113	C	Kenya			29187	GC	Swaziland
	IS13294	CB	Venezuela			29335	C	Swaziland
	IS22609	C	Sri Lanka			19676	K	Zimbabwe
	IS25548	C	Rwanda			29568	KC	Lesotho
	IS29733	GD	Zimbabwe			27912	KC	S Africa
	IS30079	DC	Zimbabwe			29468	GC	Lesotho
	IS31186	GC	Uganda			29627	DC	S Africa
	IS31446	GC	Uganda			22239	K	Botswana
	IS31557	C	Burundi			29358	K	Lesotho
	IS31651	C	Zaire			12302	C	Zimbabwe

	IS33353	C	Kenya			12945	K	Nicaragua
	IS4951	G	India			30092	DC	Zimbabwe
PG 9	IS5295	G	India			29606	K	S Africa
	IS5301	GC	India			29689	K	Zimbabwe
	IS32349	G	India			22294	K	Botswana
	IS32439	G	India			3158	K	S Africa
	IS33090	D	Honduras			29326	CB	Swaziland
	IS473	GK	USA			30231	K	Zimbabwe
PG 10	IS21512	G	Malawi			26701	CB	S Africa
	IS21645	G	Malawi			19389	C	Bangladesh
	IS23216	CB	Zambia			995	CB	USA
	IS23684	G	Mozambique			608	B	USA
	IS24139	G	Tanzania			19262	GC	Sudan
	IS24175	G	Tanzania			24462	CB	S Africa
	IS24218	G	Tanzania			14010	CB	S Africa
	IS24939	B	Zambia			14290	KD	Botswana
	IS29772	GC	Zimbabwe			24453	CB	S Africa
	IS33023	G	Tanzania			29733	GD	Zimbabwe
	IS2389	K	S Africa			27887	CB	S Africa
PG 11	IS2397	K	S Africa			22616	B	Myanmar
	IS8774	KD	S Africa			12447	DC	Sudan
	IS12302	C	Zimbabwe			12697	B	Australia
	IS12735	CB	Saudi		SG9	25732	D	Mali
	IS12945	K	Nicaragua			10969	GC	USA
	IS13782	KD	S Africa			7131	DC	Uganda
	IS13893	KC	S Africa			11026	D	Ethiopia
	IS13919	KC	S Africa			1004	D	India
	IS13971	C	S Africa			20679	GC	USA
	IS14290	KD	Botswana			25836	D	Mali
	IS19389	C	Bangladesh			12706	CB	USA
	IS19445	K	Botswana			15744	DC	Cameroon
	IS19450	GK	Botswana			28849	DC	Yemen
	IS19676	K	Zimbabwe			20195	B	Niger
	IS22239	K	Botswana			2872	CB	Egypt
	IS22294	K	Botswana			22799	D	Somalia
	IS24453	CB	S Africa			22720	D	Somalia
	IS24463	K	S Africa			20298	CB	Niger
	IS24492	K	S Africa			12804	B	Turkey
	IS26694	C	S Africa			2413	B	Iran
	IS26701	CB	S Africa		SG10	28449	GC	Yemen
	IS26737	K	S Africa			28451	GC	Yemen

	IS26749	K	S Africa			28614	DC	Yemen
	IS27887	CB	S Africa			28747	DC	Yemen
	IS27912	KC	S Africa			29091	DC	Yemen
	IS29187	GC	Swaziland			29100	DC	Yemen
	IS29233	K	Swaziland			28389	DC	Yemen
	IS29239	K	Swaziland			28141	DC	Yemen
	IS29241	KC	Swaziland			23891	D	Yemen
	IS29269	GC	Swaziland			31706	D	Yemen
	IS29304	GK	Swaziland			31714	DC	Yemen
	IS29314	DC	Swaziland			23992	C	Yemen
	IS29326	CB	Swaziland			32245	DC	Yemen
	IS29335	C	Swaziland			27034	D	Sudan
	IS29358	K	Lesotho			28313	DC	Yemen
	IS29392	K	Lesotho			27786	DB	Morocco
	IS29441	KC	Lesotho			31681	B	Algeria
	IS29468	GC	Lesotho			21863	B	Syrian
	IS29519	KC	Lesotho		SG11	1041	D	India
	IS29565	GC	Lesotho			4515	D	India
	IS29568	KC	Lesotho			4581	D	India
	IS29582	K	Lesotho			4698	D	India
	IS29606	K	S Africa			6351	D	India
	IS29627	DC	S Africa			6354	D	India
	IS29689	K	Zimbabwe			6421	D	India
	IS30092	DC	Zimbabwe			5094	D	India
	IS30231	K	Zimbabwe			32787	D	Somalia
	IS995	CB	USA			4631	D	India
Unclustered	IS1233	B	China			18039	DB	India
	IS2382	C	S Africa			12937	K	Ethiopia
	IS2413	B	Iran			17980	D	India
	IS3158	K	S Africa			4613	D	India
	IS8777	CB	Uganda			5386	D	India
	IS12447	DC	Sudan			4372	GD	India
	IS12697	B	Australia			5667	D	India
	IS14090	C	Argentina			19859	D	India
	IS20697	C	USA			4360	D	India
	IS20816	B	USA			4060	DB	India
	IS24503	B	S Africa			5919	D	India
	IS24953	GC	Zambia			3971	D	India
	IS29714	KD	Zimbabwe			8348	D	Pakistan
	IS30572	GC	Cameroon			12883	D	India

Notes: B-bicolor; C-caudatum; D-durra; G-guinea, K-kafir, and KD, KC, CB, etc. are hybrid races.
