# Supplementary material for: Genetic Structure and Linkage Disequilibrium in a Diverse, Representative Collection of the C4 Model Plant, Sorghum bicolor
Source: G3 (Bethesda). 2013 May 1;3(5):783–93. doi: 10.1534/g3.112.004861 (PMC3656726; doi:10.1534/g3.112.004861)
Supplement: Supporting Information [file supp_3.5.783_FigureS1.pdf]

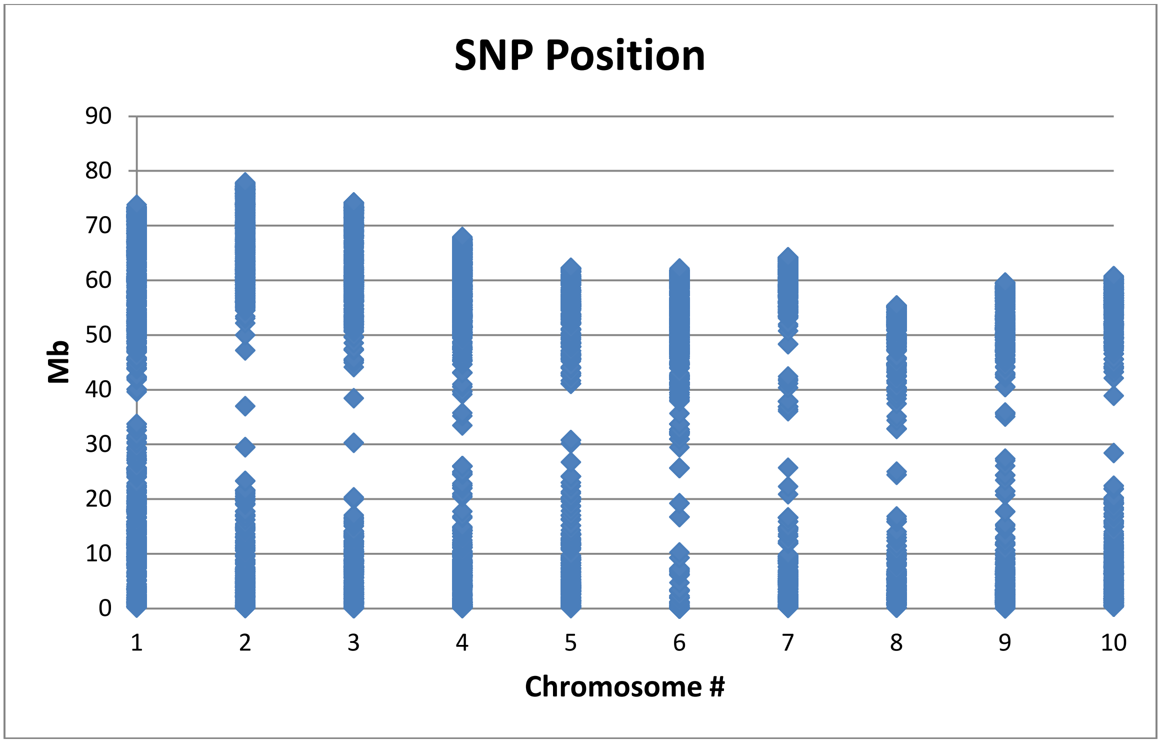

Figure S1a Distribution of SNPs utilized in this study across the 10 *Sorghum bicolor* chromosomes. The location of SNPs generated by genotyping-by-sequencing at *FseI* cut-sites are represented by blue squares. The y-axis represents the physical location of the SNPs across the sorghum genome. The SNPs are well-distributed over the 10 chromosomes and cover predominantly euchromatic regions of the genome. Regions in which SNPs are absent represent the gene-poor pericentromeric heterochromatic regions.

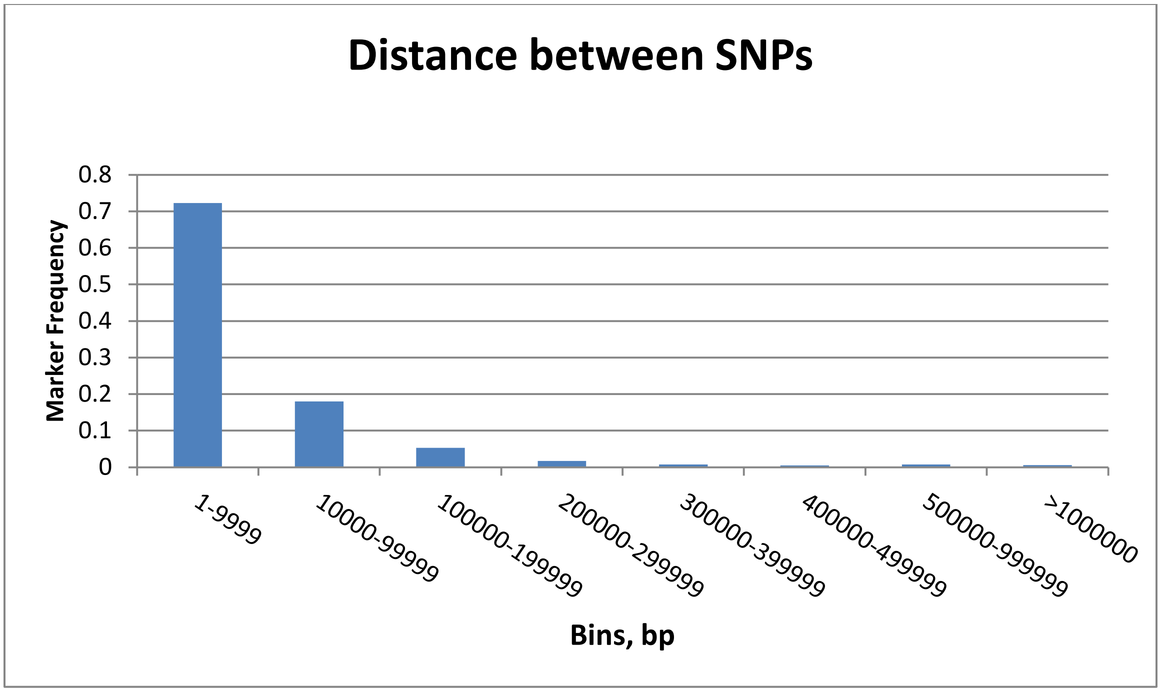

Figure S1b Distance between SNPs utilized in this study. The x-axis represents ranges of base pair distances between adjacent SNPs. The y-axis represents the percentage of total SNPs that fall within a specific range. The figure shows that 72% of the SNPs fall within 10,000 base pairs of each other.
