# Supplementary material for: Genetic Structure and Linkage Disequilibrium in a Diverse, Representative Collection of the C4 Model Plant, Sorghum bicolor
Source: G3 (Bethesda). 2013 May 1;3(5):783–93. doi: 10.1534/g3.112.004861 (PMC3656726; doi:10.1534/g3.112.004861)
Supplement: Supporting Information [file supp_3.5.783_TableS2.pdf]

Table S2 Clustering of the sorghum mini core accessions by principal component analysis and STRUCTURE using 13,390 SNP markers.

PCA groups	Accession	Race	Country		SGs	Accession	Race	Country
PG1	IS602	B	USA		SG1	23644	G	Gambia
	IS603	B	USA			25989	G	Mali
	IS608	B	USA			27697	G	SierraLeone
	IS2426	CB	Afghan			25910	G	Mali
	IS2872	CB	Egypt			24503	B	S Africa
	IS14010	CB	S Africa		SG2	10302	C	Thailand
	IS19262	GC	Sudan			19153	GC	Sudan
	IS20727	B	USA			23521	GC	Ethiopia
	IS21863	B	Syrian			23579	GC	Ethiopia
	IS22616	B	Myanmar			23586	GC	Ethiopia
	IS24462	CB	S Africa			23590	GC	Ethiopia
	IS26617	CB	Madagascar			24348	C	India
	IS27786	DB	Morocco			23514	C	Ethiopia
	IS31681	B	Algeria			17941	C	India
PG2	IS1041	D	India			4092	C	India
	IS3971	D	India			2864	C	S Africa
	IS4060	DB	India			20713	GC	USA
	IS4360	D	India			12965	C	Cuba
	IS4372	GD	India			29914	C	Zimbabwe
	IS4515	D	India			20956	DC	Indonesia
	IS4581	D	India			2379	C	S Africa
	IS4613	D	India			21083	C	Kenya
	IS4631	D	India			15170	C	Cameroon
	IS4698	D	India			29950	GC	Zimbabwe
	IS5094	D	India			9177	C	Kenya
	IS5386	D	India			7305	C	Nigeria
	IS5667	D	India			14779	C	Cameroon
	IS5919	D	India			9745	C	Sudan
	IS6351	D	India			22986	C	Sudan
	IS6354	D	India			20632	C	USA
	IS6421	D	India			20697	C	USA
	IS8348	D	Pakistan			10757	C	Chad
	IS12883	D	India			11473	C	Ethiopia
	IS12937	K	Ethiopia			15466	C	Cameroon
	IS17980	D	India			20625	DC	USA
	IS18039	DB	India			14090	C	Argentina
	IS19859	D	India		SG3	7250	G	Nigeria
	IS22799	D	Somalia			7310	G	Nigeria

	IS32787	D	Somalia			7679	G	Nigeria
PG3	IS1212	KB	China			15931	G	Cameroon
	IS1219	GB	China			16382	G	Cameroon
	IS20740	B	USA			26484	G	Benin
	IS29654	KB	China			7957	GB	Nigeria
	IS30383	CB	China			7987	G	Nigeria
	IS30400	CB	China			25089	G	Ghana
	IS30417	CB	China			15478	GC	Cameroon
	IS30443	CB	China			15945	GC	Cameroon
	IS30450	CB	China			16528	G	Cameroon
	IS30451	CB	China			30838	G	Cameroon
	IS30460	C	China			26046	G	Mali
	IS30466	CB	China			26025	G	Mali
	IS30507	CB	Korea			19975	G	Senegal
	IS30508	CB	Korea			27557	G	BurkinaFaso
	IS30533	CB	Korea			2902	CB	Nigeria
	IS30536	CB	Korea			26222	GC	Togo
	IS30562	B	Korea			10867	GC	Chad
PG4	IS22720	D	Somalia			14861	C	Cameroon
	IS23891	D	Yemen			30572	GC	Cameroon
	IS23992	C	Yemen		SG4	11619	DB	Ethiopia
	IS27034	D	Sudan			11919	DB	Ethiopia
	IS28141	DC	Yemen			13549	CB	Mexico
	IS28389	DC	Yemen			25249	DB	Ethiopia
	IS28449	GC	Yemen			25301	DB	Ethiopia
	IS28451	GC	Yemen			3121	B	USA
	IS28614	DC	Yemen		SG5	25548	C	Rwanda
	IS28747	DC	Yemen			33353	C	Kenya
	IS29091	DC	Yemen			22609	C	Sri Lanka
	IS29100	DC	Yemen			31557	C	Burundi
	IS31706	D	Yemen			31446	GC	Uganda
	IS31714	DC	Yemen			9113	C	Kenya
	IS32245	DC	Yemen			31186	GC	Uganda
PG 5	IS1004	D	India			31651	C	Zaire
	IS3121	B	USA			8916	GC	Uganda
	IS7131	DC	Uganda			9108	C	Kenya
	IS8012	B	Japan			24939	B	Zambia
	IS10969	GC	USA			31043	C	Uganda
	IS11026	D	Ethiopia		SG6	1212	KB	China
	IS11619	DB	Ethiopia			29654	KB	China
	IS11919	DB	Ethiopia			30383	CB	China

	IS12706	CB	USA			30400	CB	China
	IS12804	B	Turkey			30417	CB	China
	IS13549	CB	Mexico			30443	CB	China
	IS15744	DC	Cameroon			30450	CB	China
	IS16151	CB	Cameroon			30451	CB	China
	IS20195	B	Niger			30460	C	China
	IS20679	GC	USA			30466	CB	China
	IS20743	B	USA			30507	CB	Korea
	IS25249	DB	Ethiopia			30508	CB	Korea
	IS25301	DB	Ethiopia			30533	CB	Korea
	IS25732	D	Mali			30536	CB	Korea
	IS25836	D	Mali			30562	B	Korea
	IS28313	DC	Yemen			1219	GB	China
	IS28849	DC	Yemen			1233	B	China
PG6	IS2902	CB	Nigeria			20740	B	USA
	IS7250	G	Nigeria			16151	CB	Cameroon
	IS7310	G	Nigeria			603	B	USA
	IS7679	G	Nigeria			20727	B	USA
	IS7957	GB	Nigeria			8012	B	Japan
	IS7987	G	Nigeria			20816	B	USA
	IS10867	GC	Chad			602	B	USA
	IS15478	GC	Cameroon			2426	CB	Afghan
	IS15931	G	Cameroon			20743	B	USA
	IS15945	GC	Cameroon		SG7	23684	G	Mozambique
	IS16382	G	Cameroon			24218	G	Tanzania
	IS16528	G	Cameroon			24175	G	Tanzania
	IS19975	G	Senegal			21645	G	Malawi
	IS20956	DC	Indonesia			5295	G	India
	IS25089	G	Ghana			5301	GC	India
	IS25910	G	Mali			32349	G	India
	IS26025	G	Mali			4951	G	India
	IS26046	G	Mali			24139	G	Tanzania
	IS26222	GC	Togo			473	GK	USA
	IS26484	G	Benin			32439	G	India
	IS27557	G	BurkinaFaso			29772	GC	Zimbabwe
	IS30838	G	Cameroon			21512	G	Malawi
PG7	IS2379	C	S Africa			33023	G	Tanzania
	IS2864	C	S Africa			24953	GC	Zambia
	IS4092	C	India			23216	CB	Zambia
	IS7305	C	Nigeria			2382	C	S Africa
	IS9108	C	Kenya			30079	DC	Zimbabwe

	IS9177	C	Kenya			13294	CB	Venezuela
	IS9745	C	Sudan			29714	KD	Zimbabwe
	IS10302	C	Thailand			8777	CB	Uganda
	IS10757	C	Chad			26617	CB	Madagascar
	IS11473	C	Ethiopia			33090	D	Honduras
	IS12965	C	Cuba		SG8	2389	K	S Africa
	IS14779	C	Cameroon			2397	K	S Africa
	IS14861	C	Cameroon			8774	KD	S Africa
	IS15170	C	Cameroon			12735	CB	Saudi
	IS15466	C	Cameroon			13782	KD	S Africa
	IS17941	C	India			13919	KC	S Africa
	IS19153	GC	Sudan			13971	C	S Africa
	IS20298	CB	Niger			19450	GK	Botswana
	IS20625	DC	USA			24463	K	S Africa
	IS20632	C	USA			24492	K	S Africa
	IS20713	GC	USA			26737	K	S Africa
	IS21083	C	Kenya			26749	K	S Africa
	IS22986	C	Sudan			29233	K	Swaziland
	IS23514	C	Ethiopia			29239	K	Swaziland
	IS23521	GC	Ethiopia			29241	KC	Swaziland
	IS23579	GC	Ethiopia			29304	GK	Swaziland
	IS23586	GC	Ethiopia			29519	KC	Lesotho
	IS23590	GC	Ethiopia			29314	DC	Swaziland
	IS23644	G	Gambia			29565	GC	Lesotho
	IS24348	C	India			29582	K	Lesotho
	IS25989	G	Mali			19445	K	Botswana
	IS27697	G	SierraLeone			29392	K	Lesotho
	IS29914	C	Zimbabwe			13893	KC	S Africa
	IS29950	GC	Zimbabwe			29441	KC	Lesotho
	IS31043	C	Uganda			29269	GC	Swaziland
	IS8916	GC	Uganda			26694	C	S Africa
PG 8	IS9113	C	Kenya			29187	GC	Swaziland
	IS13294	CB	Venezuela			29335	C	Swaziland
	IS22609	C	Sri Lanka			19676	K	Zimbabwe
	IS25548	C	Rwanda			29568	KC	Lesotho
	IS29733	GD	Zimbabwe			27912	KC	S Africa
	IS30079	DC	Zimbabwe			29468	GC	Lesotho
	IS31186	GC	Uganda			29627	DC	S Africa
	IS31446	GC	Uganda			22239	K	Botswana
	IS31557	C	Burundi			29358	K	Lesotho
	IS31651	C	Zaire			12302	C	Zimbabwe

	IS33353	C	Kenya			12945	K	Nicaragua
	IS4951	G	India			30092	DC	Zimbabwe
PG 9	IS5295	G	India			29606	K	S Africa
	IS5301	GC	India			29689	K	Zimbabwe
	IS32349	G	India			22294	K	Botswana
	IS32439	G	India			3158	K	S Africa
	IS33090	D	Honduras			29326	CB	Swaziland
	IS473	GK	USA			30231	K	Zimbabwe
PG 10	IS21512	G	Malawi			26701	CB	S Africa
	IS21645	G	Malawi			19389	C	Bangladesh
	IS23216	CB	Zambia			995	CB	USA
	IS23684	G	Mozambique			608	B	USA
	IS24139	G	Tanzania			19262	GC	Sudan
	IS24175	G	Tanzania			24462	CB	S Africa
	IS24218	G	Tanzania			14010	CB	S Africa
	IS24939	B	Zambia			14290	KD	Botswana
	IS29772	GC	Zimbabwe			24453	CB	S Africa
	IS33023	G	Tanzania			29733	GD	Zimbabwe
	IS2389	K	S Africa			27887	CB	S Africa
PG 11	IS2397	K	S Africa			22616	B	Myanmar
	IS8774	KD	S Africa			12447	DC	Sudan
	IS12302	C	Zimbabwe			12697	B	Australia
	IS12735	CB	Saudi		SG9	25732	D	Mali
	IS12945	K	Nicaragua			10969	GC	USA
	IS13782	KD	S Africa			7131	DC	Uganda
	IS13893	KC	S Africa			11026	D	Ethiopia
	IS13919	KC	S Africa			1004	D	India
	IS13971	C	S Africa			20679	GC	USA
	IS14290	KD	Botswana			25836	D	Mali
	IS19389	C	Bangladesh			12706	CB	USA
	IS19445	K	Botswana			15744	DC	Cameroon
	IS19450	GK	Botswana			28849	DC	Yemen
	IS19676	K	Zimbabwe			20195	B	Niger
	IS22239	K	Botswana			2872	CB	Egypt
	IS22294	K	Botswana			22799	D	Somalia
	IS24453	CB	S Africa			22720	D	Somalia
	IS24463	K	S Africa			20298	CB	Niger
	IS24492	K	S Africa			12804	B	Turkey
	IS26694	C	S Africa			2413	B	Iran
	IS26701	CB	S Africa		SG10	28449	GC	Yemen
	IS26737	K	S Africa			28451	GC	Yemen

	IS26749	K	S Africa			28614	DC	Yemen
	IS27887	CB	S Africa			28747	DC	Yemen
	IS27912	KC	S Africa			29091	DC	Yemen
	IS29187	GC	Swaziland			29100	DC	Yemen
	IS29233	K	Swaziland			28389	DC	Yemen
	IS29239	K	Swaziland			28141	DC	Yemen
	IS29241	KC	Swaziland			23891	D	Yemen
	IS29269	GC	Swaziland			31706	D	Yemen
	IS29304	GK	Swaziland			31714	DC	Yemen
	IS29314	DC	Swaziland			23992	C	Yemen
	IS29326	CB	Swaziland			32245	DC	Yemen
	IS29335	C	Swaziland			27034	D	Sudan
	IS29358	K	Lesotho			28313	DC	Yemen
	IS29392	K	Lesotho			27786	DB	Morocco
	IS29441	KC	Lesotho			31681	B	Algeria
	IS29468	GC	Lesotho			21863	B	Syrian
	IS29519	KC	Lesotho		SG11	1041	D	India
	IS29565	GC	Lesotho			4515	D	India
	IS29568	KC	Lesotho			4581	D	India
	IS29582	K	Lesotho			4698	D	India
	IS29606	K	S Africa			6351	D	India
	IS29627	DC	S Africa			6354	D	India
	IS29689	K	Zimbabwe			6421	D	India
	IS30092	DC	Zimbabwe			5094	D	India
	IS30231	K	Zimbabwe			32787	D	Somalia
	IS995	CB	USA			4631	D	India
Unclustered	IS1233	B	China			18039	DB	India
	IS2382	C	S Africa			12937	K	Ethiopia
	IS2413	B	Iran			17980	D	India
	IS3158	K	S Africa			4613	D	India
	IS8777	CB	Uganda			5386	D	India
	IS12447	DC	Sudan			4372	GD	India
	IS12697	B	Australia			5667	D	India
	IS14090	C	Argentina			19859	D	India
	IS20697	C	USA			4360	D	India
	IS20816	B	USA			4060	DB	India
	IS24503	B	S Africa			5919	D	India
	IS24953	GC	Zambia			3971	D	India
	IS29714	KD	Zimbabwe			8348	D	Pakistan
	IS30572	GC	Cameroon			12883	D	India

Notes: B-bicolor; C-caudatum; D-durra; G-guinea, K-kafir, and KD, KC, CB, etc. are hybrid races.
